# GPR65 inhibits human trophoblast cell adhesion through upregulation of MYLK and downregulation of fibronectin via cAMP-ERK signaling in a low pH environment

**DOI:** 10.1186/s12964-023-01249-3

**Published:** 2023-09-18

**Authors:** Jia Mao, Ying Feng, Yayun Zheng, Yaqiu Gao, Linyu Zhang, Xinrui Sun, Yilun Wu, Xiaofeng Zhu, Fang Ma

**Affiliations:** 1 Key Laboratory of Bio-Resource and Eco-Environment of Ministry of Education, College of Life Sciences, Sichuan University, Chengdu, 610064 Sichuan China; 2 Center for Translational Medicine, Key Laboratory of Birth Defects and Related Diseases of Women and Children (Sichuan University), Ministry of Education, West China Second University Hospital, Sichuan University, Chengdu, 610041 Sichuan China; 3 Department of Histology, Embryology and Neurobiology, West China School of Basic Medical Sciences & Forensic Medicine, Sichuan University, Chengdu, 610041 Sichuan China; 4 Department of Obstetrics and Gynecology, West China Second University Hospital, Sichuan University, Chengdu, 610041 Sichuan China

**Keywords:** GPR65, Adhesion, Trophoblast cells, Early pregnancy loss, cAMP-ERK signaling pathway

## Abstract

**Background:**

Extravillous trophoblasts (EVTs) are essential cells during the formation of the placenta, with the major function of invading the maternal decidua, anchoring the developing placenta to the uterus, remodeling uterine arteries, and regulating immune responses to prevent rejection. During early pregnancy, the decidua undergoes a hypoxic and acidic microenvironment, which has been shown to participate in tumor cell migration, invasion, growth, and angiogenesis. Nevertheless, the mechanisms by which EVTs sense and respond to the acidic microenvironment, thereby executing their functions, remain poorly understood.

**Methods:**

The effects of G protein-coupled receptor 65 (GPR65) on cell adhesion and other cellular functions were tested using JAR spheroids, mouse blastocysts, and HTR-8/SVneo cells. Specifically, we employed HTR-8/SVneo cells for gene overexpression and silencing to investigate the underlying mechanism of GPR65's impact on trophoblast cell function under acidic conditions. Additionally, villus tissue samples obtained from early pregnancy loss patients were utilized to explore the potential association between GPR65 and its related signaling pathway molecules with the disease.

**Results:**

This study identified GPR65 expression widely in trophoblasts, with the highest level in EVTs. Importantly, optimal GPR65 levels are required for maintaining normal adhesion, migration, and invasion, whereas overexpression of GPR65 inhibits these functions by activating the cAMP-ERK signaling pathway, upregulating myosin light chain kinase (MYLK) and MYLK3 expression, and subsequently downregulating fibronectin. Consistently, elevated expression of GPR65, MYLK, and MYLK3 is observed in patients suffering from early pregnancy loss.

**Conclusions:**

This work offers insights into the suppressive effects of GPR65 on EVT function under acidic conditions and highlights a putative target for therapeutic intervention in early pregnancy complications.

Video Abstract

**Supplementary Information:**

The online version contains supplementary material available at 10.1186/s12964-023-01249-3.

## Background

Placental insufficiency is a common pathology that can cause adverse pregnancy outcomes, such as pregnancy loss and recurrent miscarriage [1]. During pregnancy, cytotrophoblasts (CTBs) possess both proliferative and differentiation properties, enabling them to differentiate into syncytiotrophoblasts (STBs) and extravillous cytotrophoblasts (EVTs) [2]. STBs are initialized by CTB fusion and have no obvious cell boundaries. They play a role in nutrition, metabolism, and endocrine functions. EVTs infiltrate the decidua and participate in the remodeling of uterine spiral arteries [3]. Before maternal placental blood circulation is established, the blastocyst resides in a hypoxic environment [4]. Hypoxia triggers an increase in cell glycolysis, producing and releasing lactate, and results in an extracellular acidic environment [5]. On the one hand, the physiological hypoxia-induced low pH environment plays a crucial role in regulating trophoblast cell differentiation and invasion. On the other hand, excessive or sustained pathological hypoxic and an acidic microenvironment can impair placental development, affecting pregnancy outcomes. Previous studies have shown that the NLRP7 inflammasome is upregulated in trophoblast cells under hypoxic conditions and regulates cell proliferation, migration, and invasion. It was upregulated in the placenta with fetal growth restriction, suggesting that NLRP7 may be involved in the pathogenesis of fetal growth restriction (FGR) [6]. Additionally, hypoxia leads to the downregulation of CK2 enzyme, and inhibiting its enzymatic activity reduces trophoblast cell invasion, migration, and syncytialization. In the placenta of preeclampsia (PE) patients, CK2 enzymatic activity and expression levels are significantly upregulated, indicating an association between abnormal CK2 expression and placental development and pregnancy outcomes [7]. A pathological hypoxic and acidosis microenvironment negatively affects placental and fetal development, possibly leading to FGR, PE, and intrauterine death [8]. Therefore, investigating the regulation of trophoblast cell function in the acidic microenvironment is essential to elucidate the adaptive and functional changes of trophoblast cells in both physiological and pathological acidotic microenvironments.

G protein-coupled receptor 65 (GPR65) is a pH sensor on the cell membrane [9]. It was first found in apoptotic thymocytes as an orphan G protein-coupled receptor, and high expression of GPR65 and other pH-sensing GPCRs can be found in various tumors [10]. By sensing extracellular H^+^ in acidic conditions and activating the cAMP signaling pathway [11], GPR65 induces stimulation in RhoA (a small molecule G protein), actin shift, and stress fiber formation at low pH levels [12]. Overexpression of GPR65 in tumor cells can promote cell survival and growth in an acidic environment and induce tumor development in mice. This may serve as a measure to prevent tumor cells from acidosis by increasing the expression of acid receptors [13]. However, there are studies indicating that GPR65 exhibits tumor suppressor functions in certain tumors. For example, the expression of GPR65 in human lymphoma samples is significantly reduced compared with that in normal lymphoid tissues, indicating that GPR65 has a potential tumor suppressor function in lymphoma [14]. Additionally, GPR65 has been found to be markedly downregulated in hematological malignancies, leading to reduced tumor cell proliferation specifically under conditions of pH 7.4 and severe acidosis (pH 6.4) [15]. GPR65 is also found in the placenta, but its specific function has not been reported. Since the migration and invasiveness of trophoblast cells and malignant tumor cells share similarities, they may involve the same cellular processes and signaling pathways [16, 17].

To prove this inference, we investigated the function of GPR65 in human trophoblasts in a low pH environment. The results demonstrated that the expression of GPR65 exerted inhibitory effects on various functions, including the growth and adhesion of JAR spheroids and mouse blastocysts. It is noteworthy that GPR65 exhibited predominant expression in EVTs within placental villi, where it was found to inhibit crucial cellular processes such as adhesion, migration, invasion, and endothelial-like tube formation in HTR-8/SVneo cells. Furthermore, GPR65 was observed to influence the organization of the actin cytoskeleton and impede microtubule formation in HTR-8/SVneo cells. The mechanism by which GPR65 inhibits cell adhesion is related to the activation of the cAMP and ERK pathways with higher expression levels of myosin light chain kinase (MYLK) and myosin light chain kinase 3 (MYLK3) and lower expression of fibronectin (FN). Remarkably, consistent with these findings, we observed upregulation of GPR65, MYLK, and MYLK3 in the villous tissue obtained from cases of early pregnancy loss. In general, these findings provide substantial evidence supporting a critical role for GPR65 in regulating placental development and offer valuable insights into the underlying mechanisms contributing to adverse pregnancy outcomes, including early pregnancy loss.

## Materials and methods

### Human tissue samples

This study collected chorionic tissue from the first trimester from both normal pregnancies and pregnancy loss carried out legally (normal group, *n* = 15; pregnancy loss group, *n* = 15). RNA isolation was performed on some samples, while others were embedded in formalin-fixed paraffin wax for tissue sections of 3 μm.

All tissues were collected with informed consent at the operating room of the West China Second University Hospital, Sichuan University. The clinical characteristics of all participants are presented in Table S1. The protocol for sample collection was approved by the Ethics Committee of the West China Second University Hospital, Sichuan University (2018018).

### Cell culture

Human endometrial epithelial cells (Ishikawa cells), HTR-8/SVneo cells, and JAR cells were cultured in phenol red Roswell Park Memorial Institute (RPMI) 1640 medium (Gibco, C22400500BT), and 293T cells were cultured in DMEM (Gibco, C11995500BT). The cells were incubated at 37°C with 5% CO_2_. The cultures were supplemented with 10% fetal bovine serum (Gibco, A3160802) and 1% penicillin–streptomycin (Solarbio, P1400).

### Immunohistochemistry (IHC)

The paraffin sections were deparaffinized in xylene and rehydrated through graded alcohols to water. Next, antigen retrieval was performed by heating the sections in sodium citrate buffer at a high temperature for 10 min. Endogenous peroxidase activity was blocked by treating the sections with 3% H_2_O_2_. Then, nonspecific binding was blocked with 3% BSA (BIOFROXX, 4240GR100) for 1 h. The sections were then incubated overnight at 4°C with the appropriate primary antibody. The following day, corresponding secondary antibodies were added and incubated for 1 h at room temperature. After developing the color with DAB (ZSGB-BIO, ZLI-9017), the sections were counterstained with hematoxylin for 1–3 min. Finally, the sections were dehydrated and mounted with a xylene-based mounting medium, and images were captured using a microscope (Nikon).

The following antibodies were used: GPR65 (Abcam, ab188907, 1:100) and HLA-G (Santa Cruz, sc-21799, 1:100). MYLK (Proteintech, 21642–1-AP, 1:100), MYLK3 (Proteintech, 21527–1-AP, 1:100).

### Virus production

To generate lentiviral vectors, we employed two plasmids: pLVX-Puro-GPR65 and pLVX-EGFP-Puro-*Gpr65*. These plasmids were cotransfected into 293T cells along with packaging plasmids pMD2.G and psPAX2. Subsequently, we collected the resulting viral liquid. The pLVX-Puro-GPR65 plasmid was utilized to generate stable transgenic HTR-8/SVneo and JAR cell lines, which overexpress GPR65. In addition, the pLVX-EGFP-Puro-*Gpr65* plasmid was used to overexpress GPR65 in mouse blastocysts. The expression of GPR65 was confirmed by fluorescence microscopy, as the pLVX-EGFP-Puro plasmid carries the eGFP signal.

### siRNA and plasmid transfections

Cells were transfected with siRNA using Lipofectamine 3000 (Invitrogen, L3000015) according to the manufacturer's instructions. siRNA target sequences are listed in Table S2.

### Flow cytometry

For flow cytometry, treated cells were collected into microtubes and fixed with 4% paraformaldehyde for 10 min. Next, the cells were blocked using 3% BSA for 1 h. Following the blocking step, the cells were stained with GPR65 antibody at a dilution of 1:500 and incubated at room temperature for 1 h. Subsequently, the cells were washed once with PBS and then incubated with a corresponding secondary antibody (PE Donkey anti-rabbit IgG, Biolegend, 406421) for 1 h at room temperature. Finally, the cells were analyzed using a Cytoflex flow cytometer for detection and analysis.

### Adhesion and migration/invasion assays

HTR-8/SVneo cells were first counted after trypsinization, resuspended in complete medium at different pH values, and then subjected to different experiments.

For the adhesion assay, a 96-well plate was prepared by adding Matrigel (BD, 356234), followed by adding 3 × 10^4^ cells in each well. The cells were cultured for 2 h.

In the migration and invasion experiments, the cells in each group were treated with 1 μg/mL mitomycin C (Sigma, M5353) for 24 h. After trypsinization, the cells were resuspended in media with different pH values for migration and invasion experiments in transwell chambers.

For the migration assay, we used a small chamber that was filled with 5 × 10^4^ cells in the upper layer and serum-free medium. The cells were cultured for 8 h.

For the invasion assay, Matrigel was added to the small chamber, and 8 × 10^4^ cells were added to each well. The cells were cultured for 8 h.

Upon completion of the experiments, cells were stained with 0.1% crystal violet for 10 min, and photographs were taken using an inverted microscope. The cells were then counted using ImageJ, and normalization was carried out by using the control treatment group.

### JAR spheroid formation and 3D invasion model

Ultralow adhesion culture plates were utilized to produce JAR spheroids [18]. JAR cells were seeded onto ultralow adhesion culture plates (LABSELECT, 11318) at 4 × 10^3^ cells/well. Following 24 h of incubation in a cell culture incubator, the cells aggregated and formed spheroids of various sizes. These spheroids were then filtered through 70 μm and 100 μm cell strainers (WHB) and repeatedly washed with PBS to obtain JAR spheroids with diameters of 70–100 μm. The JAR spheroids were resuspended in cell culture medium at pH 6.5 and 7.6.

For the JAR spheroid attachment model, JAR spheroids were suspended on a bed of Ishikawa cells at a density of 30 spheroids per well to create the JAR spheroid attachment model [19]. After 4 h of incubation, the culture plates were gently shaken on a shaker at 140 rpm for 10 min to remove unadhered JAR spheroids. Trophoblast and endometrial epithelial cell attachment abilities were assessed by determining the percentage of successfully adhered JAR spheroids.

For the JAR microdrop growth assay [20], a mixture of Matrigel and JAR spheroid suspension was created at a 1:10 ratio. Then, 35 μL of the suspension was added to a 24-well plate for cell suspension culture, and microscopy images were captured at 4 and 9 h after seeding.

In the 3D invasion model, an extracellular matrix (ECM) layer was prepared by adding 50 μL of a mixture of Matrigel and collagen at a ratio of 1:5 to the wells of a 96-well plate [21]. Later, the bottom layer of the plate was seeded with Ishikawa cells stained with a Cell Plasma Membrane Staining Kit with DiO (Beyotime, C1993S). Next, a suspension of JAR spheroids stained with the Cell Plasma Membrane Staining Kit with DiD (Beyotime, C1995S) was seeded onto the ECM layer. The plate was then incubated at 37 °C and photographed using a confocal microscope at 0 h and 72 h to determine the distance of JAR spheroids invading the ECM layer.

### Animals and lentiviral transduction

Animal studies in this research were approved by the Ethics Committee of the West China Second University Hospital, Sichuan University (2021044). We mated healthy adult female ICR mice aged 6–8 weeks with fertile males to initiate pregnancy naturally. The morning of verifying a vaginal plug was designated as day 1 of pregnancy. On Day 4, blastocyst embryos were collected from the female mice. The zona pellucida was removed using acidic Tyrode's solution (Sigma, T1788).

### Mouse blastocyst adhesion assay and trophoblast outgrowth analysis

Mouse blastocysts were incubated with *Gpr65*-lentivirus (LV) or control plasmid LV (CTRL-LV) solution. Subsequently, they were cocultured at 37°C in a 5% CO_2_ atmosphere with or without confluent monolayers of Ishikawa cells. After 48 h of incubation, the growth of the blastocysts was observed under an inverted microscope. Confluent monolayers of Ishikawa cells were cultured in 96-well plates, and 5–8 mouse embryos were added to each well. Each experiment used 10–15 mouse blastocysts for a given condition. The attachment of the embryos was examined under an inverted microscope.

### Fluorescence imaging

HTR-8/SVneo cells were pressed on glass slides and fixed in 4% paraformaldehyde for 15 min. The cells were permeabilized with 0.3% Triton X 100 for 10 min and blocked in a 1% bovine serum albumin solution for 1 h. α-Tubulin antibodies (Proteintech, 11224–1-AP) were added and incubated overnight at 4°C. After washing 3 times in PBS with 0.1% Tween-20, the cells were incubated with an Alexa Fluor 568-conjugated antibody (A-11001, Invitrogen) mixture for 1 h at room temperature. Nuclei were stained with DAPI (Solarbio, 28718–90-3, S10 μg/ml) for 15 min, and the cells were washed 3 times before being mounted on glass slides. Microanalysis was conducted using a Leica Stellaris 5 confocal microscope.

Cytoskeleton staining was performed by fixing the cells and staining them with Actin-Tracker Green (C2201S, Beyotime) for 30 min at room temperature in the dark.

### Endothelial-like tube formation

We used a 96-well plate coated with Matrigel, which provides the necessary substrate to study endothelial-like tube formation in vitro. First, HTR-8/SVneo cells were counted after trypsinization and resuspended in complete medium at different pH levels (pH 6.5 and pH 7.6). Next, 3 × 10^4^ cells were inoculated in each well of the coated 96-well plate containing 50 μL of Matrigel. The cells were cultured for 3 h, and digital images were captured using a light microscope. Finally, the branch points and total length of the formed tubes were measured using ImageJ software.

### RNA sequencing and analysis

The control HTR-8/SVneo cells and GPR65-overexpressing HTR-8/SVneo cells were treated with pH 6.5 and pH 7.6 complete medium for 24 h. RNA sequencing was performed on a PromethION platform at Biomarker Technology Company (Beijing, China). Differential expression analysis of two groups (three biological replicates per condition) was performed using the DESeq2 R package (1.20.0). The threshold for significantly differential expression was set as genes showing a fold change ≥ 2 and a *P* value < 0.05. The transcriptome obtained in this study has been uploaded to the NCBI database, and the research project number is PRJNA953774.

### ELISA for cAMP

After treatment with IBMX (Beyotime, SC0195) at a final concentration of 0.5 mM for 15–30 min, the cells were further treated with serum-free medium at pH 6.5 and pH 7.6 for 30 min. Next, 0.1 M HCl was added to collect the cells into a centrifuge tube. The levels of cAMP secretion in the cell supernatants were analyzed using an ELISA kit following the manufacturer's instructions (581001; Cayman). Briefly, the cell extract was added to a 96-well cover sheet, followed by the corresponding cAMP AChE Tracer and cAMP ELISA Antiserum, and incubated at 4 °C for 18 h. After washing with Wash Buffer 5 times, Tracer was added to develop color for 90 min. The plate was then read at a wavelength of 412 nm, and the cAMP concentration was calculated according to the standard curve.

### RT‒qPCR

RNA was extracted from the cells using TRIzol (Ambion, 15596026) following the manufacturer's instructions. Subsequently, cDNA was synthesized using a reverse transcription kit. Primers were designed and synthesized by Qingke and are listed in Table S3. SYBR Green mix (Invitrogen, 11744500) was used for qPCR. qPCR was performed on a Bio-Rad instrument using the following procedure: amplification was performed at 95°C for 2 min, followed by 39 cycles of 95°C for 15 s and 60°C for 30 s. GAPDH was used as an internal reference for data normalization, and the 2^−ΔΔCT^ method was used for quantitative analysis.

### Western blotting

The cells were lysed using RIPA lysis buffer supplemented with a protease inhibitor cocktail (CWBIO, CW2200). After high-speed centrifugation, the protein supernatant was collected and quantified with a BCA kit (Biosharp, BL521A). Then, 20 μg of protein was loaded onto a 10% SDS* − *PAGE gel for electrophoresis at 90 V for 30 min, followed by 120 V for 1 h. After transfection for 1 h, nitrocellulose membranes were blocked with TBST containing 5% fat* − *free dry milk for 1 h. The primary antibody corresponding to the target protein was added and incubated overnight at 4°C. The next day, we added the corresponding secondary antibodies and incubated them for 1 h at room temperature. For color development, we used ECL luminescent liquid (Biosharp, BL523B) and an exposure instrument for imaging. We quantitatively analyzed protein expression by using GAPDH as a housekeeping gene.

The following antibodies were used for Western blotting: ERK (Millipore, 2036119, 1:1000), pERK (Millipore, 2225197, 1:1000), MYLK (Proteintech, 21642–1-AP, 1:1000), MYLK3 (Proteintech, 21527–1-AP, 1:1000), and GAPDH (ABclonal, AC033, 1:10000). HRP goat anti-rabbit IgG (ABclonal, AS014, 1:1000) and HRP goat anti-mouse IgG (ABclonal, AS003, 1:1000) were used.

### Statistical analysis

Statistical analysis was performed using GraphPad Prism 9. Data are presented as the mean ± standard error of the mean (SEM) from at least three independent experiments. The significance of differences between treatments and corresponding controls was determined using an independent-sample t test. Statistical significance is indicated: *P < 0.05, **P < 0.01, and ***P < 0.001.

## Results

### GPR65 inhibits the adhesion, growth, and invasion of JAR spheroids and mouse blastocysts

**Fig. 1:**
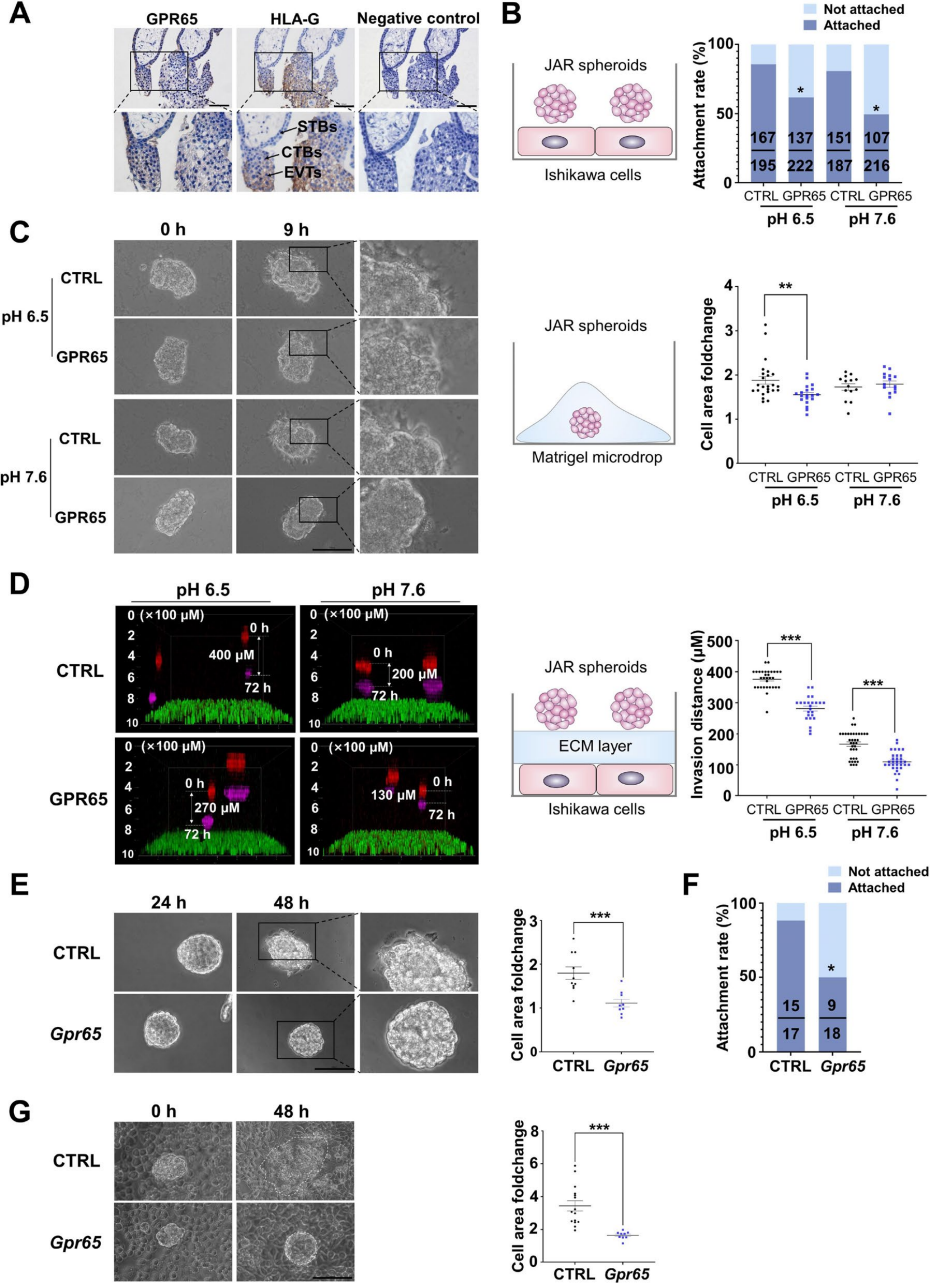
GPR65 inhibits the adhesion, growth, and invasion of JAR spheroids and mouse blastocysts. **A** IHC staining of GPR65 in the first-trimester human villus tissue at 7 weeks of gestation (WG) shows CTBs and STBs staining positively for GPR65, with higher expression in EVTs. **B** The effect of GPR65 on JAR spheroid attachment to Ishikawa cells. **C** GPR65 decreased the growth size of JAR spheroids in Matrigel microdroplets. **D** GPR65 inhibited the invasion of JAR spheroids in the ECM layer. **E** The effect of Gpr65 on mouse blastocyst growth. **F**, **G** Gpr65 decreased the attachment and growth of mouse blastocysts cocultured with Ishikawa cells. Scale bars: 100 μm. Results are presented as the mean ± SEM of at least three independent experiments, and the statistical analysis is shown: *P < 0.05, **P < 0.01, and ***P < 0.001

GPR65 was localized in the villous tissue from the 7th week of gestation through immunohistochemistry (IHC) staining and colocalized with EVTs through HLA-G staining [22]. The results showed that GPR65 was localized in CTBs, STBs, and EVTs, with more significant expression in EVTs (Fig. 1A). This indicates its participation in the differentiation, adhesion, migration, and invasion of trophoblast cells in early pregnancy. To investigate the role of GPR65 in trophoblast cell adhesion and invasion, JAR (human choriocarcinoma cell line) spheroids and mouse blastocysts were utilized as implantation models [23]. In addition, lentiviral supernatant was used to overexpress GPR65 in JAR cells and mouse blastocysts. Subsequently, we evaluated the effects on cell behavior in various in vitro models.

In the JAR cell model, the construction of JAR cells overexpressing GPR65 was confirmed by RT‒qPCR and immunofluorescence (Fig. S1). JAR spheroids were inoculated on Ishikawa cells to examine their adhesion to the endometrial epithelium. GPR65 significantly reduced the adhesion ability of JAR spheroids to Ishikawa cells at pH 6.5 and 7.6 (Fig. 1B). By inoculating JAR spheroids in Matrigel microdroplets and recording their growth, we found that GPR65 inhibited the growth ability of JAR spheroids in the Matrigel microdroplets (Fig. 1C). A 3D ECM microenvironment was developed to function as a substrate for JAR spheroid invasion. Specifically, we inoculated spheroids on the top layer of the substrate and used confocal microscopy to photograph their invasion location in the ECM layer after 72 h. The results showed that overexpression of GPR65 significantly reduced the invasion distance of JAR spheroids in the ECM layer at pH 6.5 and pH 7.6 compared with the control group (Fig. 1D). Notably, the infiltration of cells was found to be more significant at pH 6.5 than at pH 7.6. These results suggest that GPR65 inhibits trophoblast cell adhesion, invasion, and growth.

**Fig. 2:**
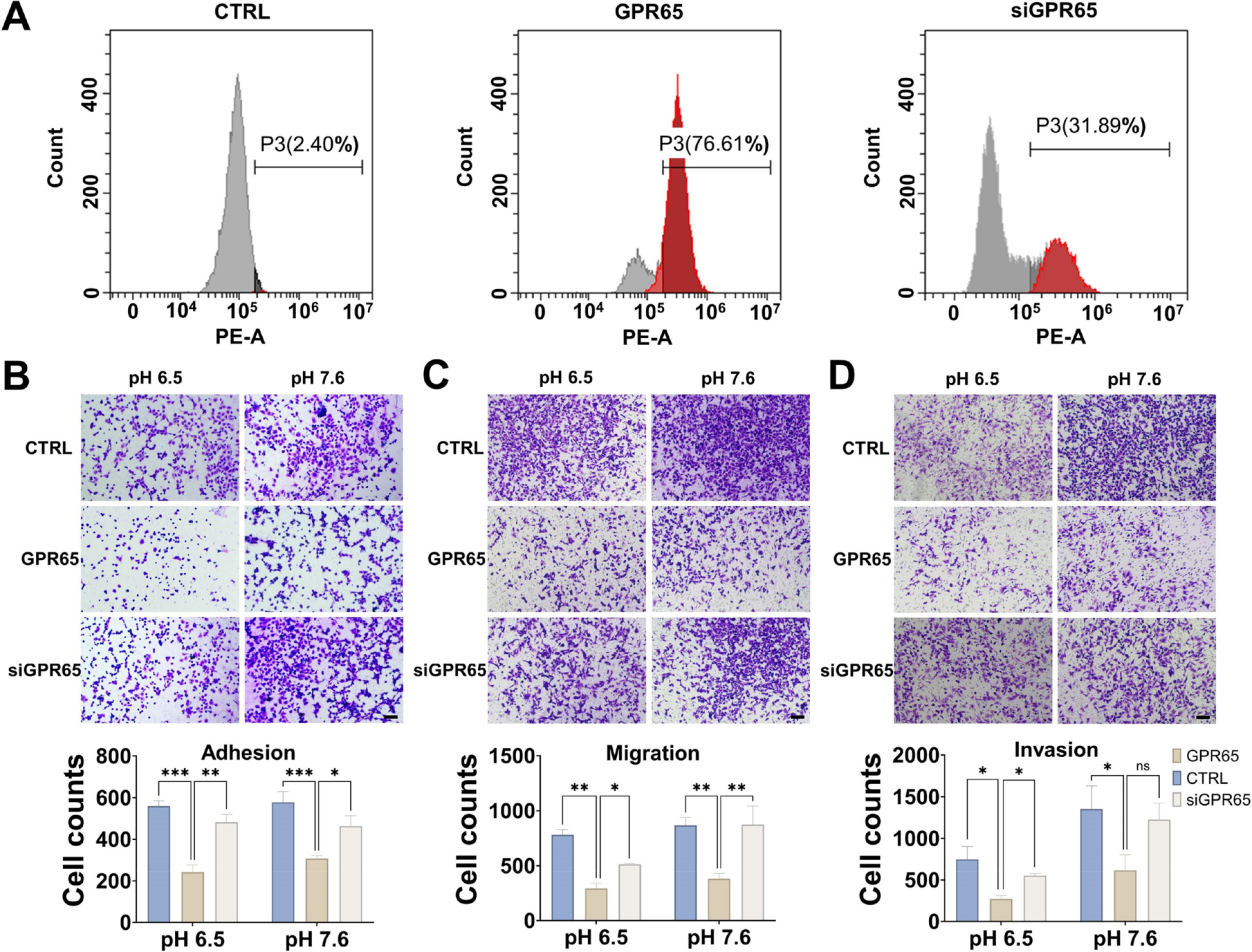
GPR65 reduces the adhesion, migration, and invasion of HTR-8/SVneo cells. **A** The expression level of GPR65 was detected by flow cytometry in GPR65-overexpressing and GPR65-silenced HTR-8/SVneo cells. **B**-**D** GPR65 significantly attenuated adhesion, migration, and invasion in HTR-8/SVneo cells. All adhered, invaded, and migrated cells were stained and photographed. After treatment, the fold change in cell adhesion, invasion, and migration capacity was estimated by counting the adhered, migrated, and invaded cells with staining. Scale bars: 100 μm. Results are presented as the mean ± SEM of at least three independent experiments, and the statistical analysis is shown: *P < 0.05; **P < 0.01; ***P < 0.001

In the blastocyst model, the efficiency of *Gpr65* transfection into mouse blastocysts was confirmed by immunofluorescence detection of the GFP signal carried on the plasmid (Fig. S1). After culturing *Gpr65*-overexpressing blastocysts for 48 h, *Gpr65* inhibited the outward expansion of trophoblast cells, which reduced their growth area, suggesting that *Gpr65* inhibits the motility of trophoblast cells in blastocysts (Fig. 1E). Furthermore, blastocysts were inoculated on monolayers of Ishikawa cells, and their adhesion rates were evaluated after 48 h. The results showed that *Gpr65* inhibited the adhesion of blastocysts to Ishikawa cells (Fig. 2F). The adherent cells continued growing, and the overexpression of *Gpr65* reduced the growth area of blastocysts and hampered their development (Fig. 2G). These findings imply that GPR65 negatively affects blastocyst colonization and development.

### GPR65 reduces the adhesion, migration, and invasion of HTR-8/SVneo cells under low pH conditions

Based on the high expression level of GPR65 in extravillous trophoblasts (EVTs), we selected HTR-8/SVneo cells, which exhibit EVT-like characteristics, as a model to investigate the function of GPR65. We established a stable HTR-8/SVneo cell line with high GPR65 expression using lentivirus transfection and then silenced GPR65 expression with siRNA transfection. Flow cytometry confirmed the significant upregulation and downregulation of GPR65 expression (Fig. 2A). We artificially induced an acidic environment under pH 5.0, 6.0, 6.5, and 7.6 conditions and observed the effect of GPR65 on HTR-8/SVneo cell adhesion. The results indicate a significant reduction in adhesion at pH 6.5 with GPR65 compared to the control (Fig. S3). Since the pH value of the placenta in the first trimester of pregnancy cannot be detected, the pH value with the most obvious inhibitory function was chosen to simulate the extracellular acidic environment to study the function of GPR65. We further examined the effects of GPR65 on the adhesion, migration, and invasion functions of HTR-8/SVneo cells at pH 6.5 and 7.6. The results showed that upregulation of GPR65 inhibited these functions, while downregulation of GPR65 promoted them (Fig. 1B-D). Furthermore, the findings from the wound healing experiments were consistent with the transwell migration results (Figure S4). GPR65 inhibited cell proliferation at both pH 6.5 and 7.6. Notably, the wound healing rate was significantly higher at pH 7.6 compared to pH 6.5. GPR65 did not affect the proliferation of HTR-8/SVneo cells at pH 6.5 and pH 7.6, but significantly enhanced the proliferation of HTR-8/SVneo cells at pH 7.6 compared to pH 6.5 (Fig. S5). This phenomenon is consistent with the upregulation trends in adhesion, migration, and invasion at pH 7.6, suggesting that the level of cell function at pH 7.6 may have an upregulation trend due to increased cell proliferation.

### GPR65 affects cytoskeleton formation, microtubule polymerization, and endothelial-like tube formation

**Fig. 3:**
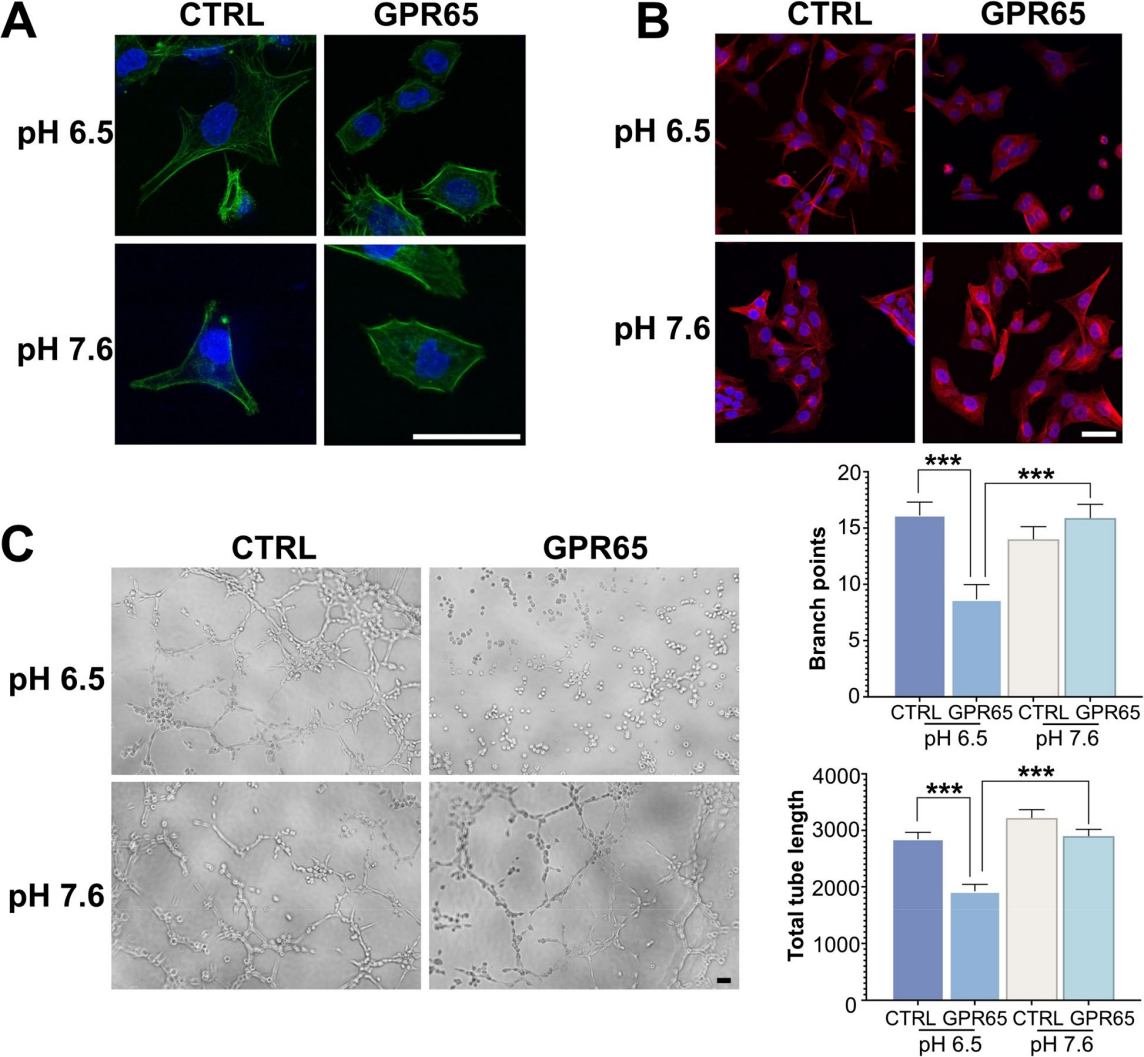
Effects of GPR65 upregulation on the actin cytoskeleton and cell endothelial-like tube formation. **A** Visualization of actin organization via immunofluorescence. **B** α-Tubulin was detected by immunofluorescence and confocal microscopy. **C** Representative images of endothelial-like tube formation after 3 h showed a tubule network. The left panels show representative images from the endothelial-like tube formation assays. The right panel shows combined quantitative results from image analysis of branch points and total tube length. Scale bars: 50 μm. Results are presented as the mean ± SEM of at least three independent experiments, and the statistical analysis is shown: *P < 0.05, **P < 0.01, and ***P < 0.001

GPR65 is known to function as an acid receptor and can stimulate stress fiber formation and Rho activation in an extracellular acidic environment [12]. The upregulation of GPR65 in HTR-8/SVneo cells led to a significant reduction in linear actin filament length compared to the control (Fig. 3A), indicating that GPR65 affects the actin cytoskeleton and consequently alters cell adhesion and migration. Moreover, we investigated the morphological changes in microtubules in HTR-8/SVneo cells using immunofluorescence and observed that microtubules in the control group were filamentous and uniformly distributed at pH 6.5. It is worth noting that microtubule aggregation was disrupted after GPR65 overexpression (Fig. 3B). This implies that cellular GPR65 may regulate cell migration by inhibiting microtubule formation.

During the early stage of placental development, establishing a spiral artery by trophoblast cells is crucial to establishing blood circulation [24]. A well-formed cytoskeleton facilitates the formation of blood vessels and the construction of spiral arteries [25]. The results revealed that GPR65 overexpression led to a reduced potential of HTR-8/SVneo cells to form a tubular network and instead resulted in the aggregation of cells into clumps, particularly at pH 6.5 (Fig. 3C). Therefore, GPR65 inhibits the potential of HTR-8/SVneo cells to form blood vessels.

### Transcriptome analysis of GPR65 affecting HTR-8/SVneo cells at low pH

**Fig. 4:**
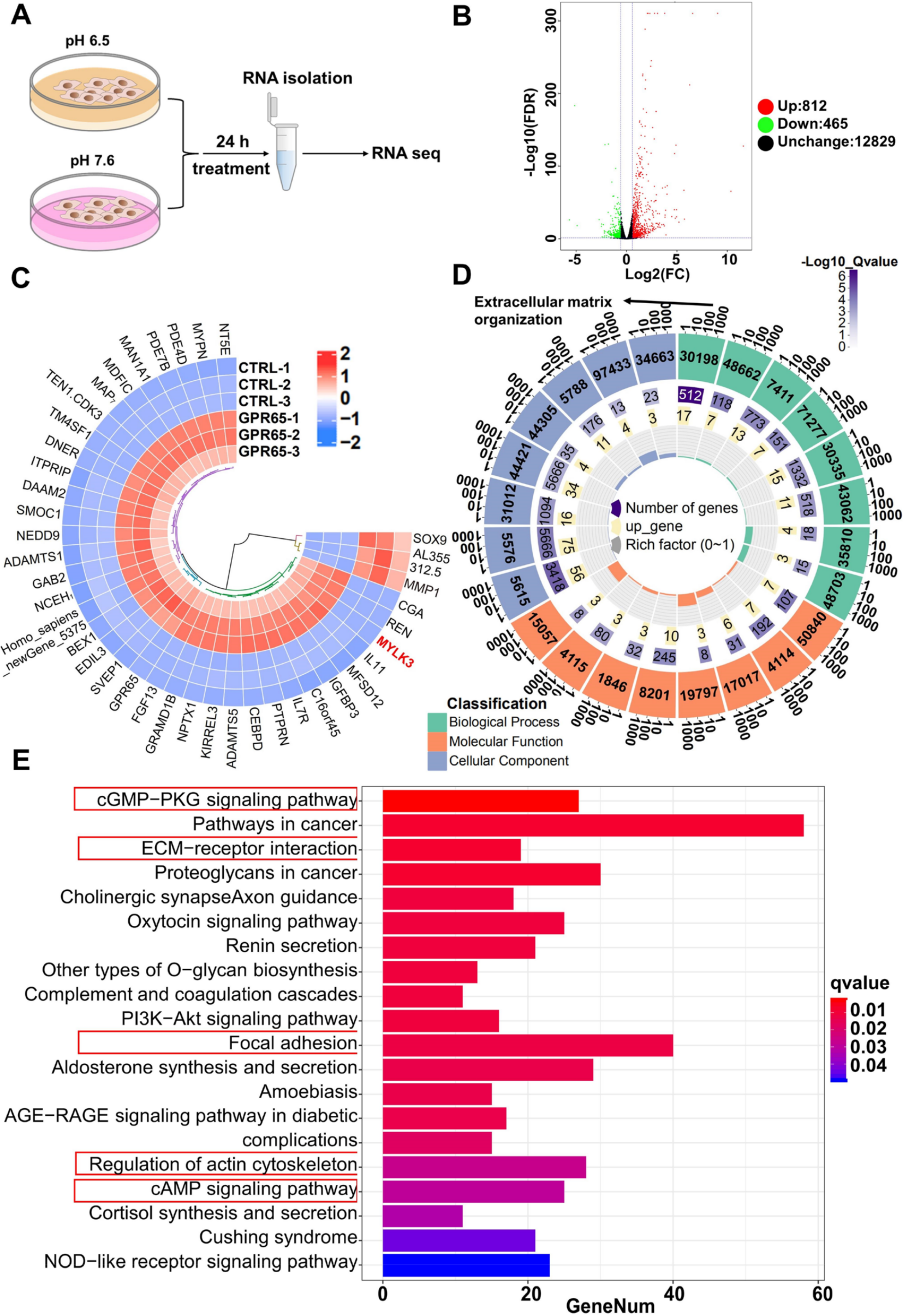
Transcriptome analysis of the effect of GPR65 on HTR-8/SVneo cells. **A** A schematic representation of the RNA-seq experiment. **B** A volcano plot of RNA-seq data from GPR65-overexpressing HTR-8/SVneo cells and control cells. When detecting differentially expressed genes (DEGs), a fold change ≥ 2 and FDR < 0.05 were the screening criteria. **C** A cluster analysis of all the DEGs was obtained from RNA-seq data. Each column represents a DEG, while each loop represents a sample. The blue and red color gradients indicate decreases and increases in transcript abundances, respectively. **D** Gene Ontology (GO) categories are presented in circle charts. **E** The top 20 significantly enriched pathways from KEGG pathway analysis of DEGs

To further investigate the molecular mechanism involved in the regulatory function of GPR65 in cells, RNA sequencing analysis was performed on GPR65-overexpressing HTR-8/SVneo cells (Fig. 4A). The analysis revealed that the low pH-treated control and GPR65-overexpressing groups had the highest number of differentially expressed genes, with 812 upregulated genes and 465 downregulated genes (Fig. 4B). A heatmap for clustering based on gene expression levels displayed a distinct separation between the two groups (Fig. 4C). The GO enrichment analysis identified an association between GPR65 and ECM (Fig. 4D). Detailed GO analysis results are illustrated in Fig. S6. Furthermore, KEGG pathway enrichment analysis showed that GPR65 was associated with classical signaling pathways related to GPCR receptors, such as the cGMP-PKG signaling pathway, PI3K-Akt signaling pathway, and cAMP signaling pathway, in addition to ECM receptors, cell motility-related pathways, the actin skeleton and focal adhesion, and other signaling pathways related to cell motility (Fig. 4E). In summary, it is speculated that GPR65 regulates the cytoskeleton and ECM through the classical GPCR signaling pathway, thereby affecting cell motility.

### GPR65 regulates HTR-8/SVneo cell adhesion via the cAMP-ERK-MYLK axis in a low pH environment

**Fig. 5:**
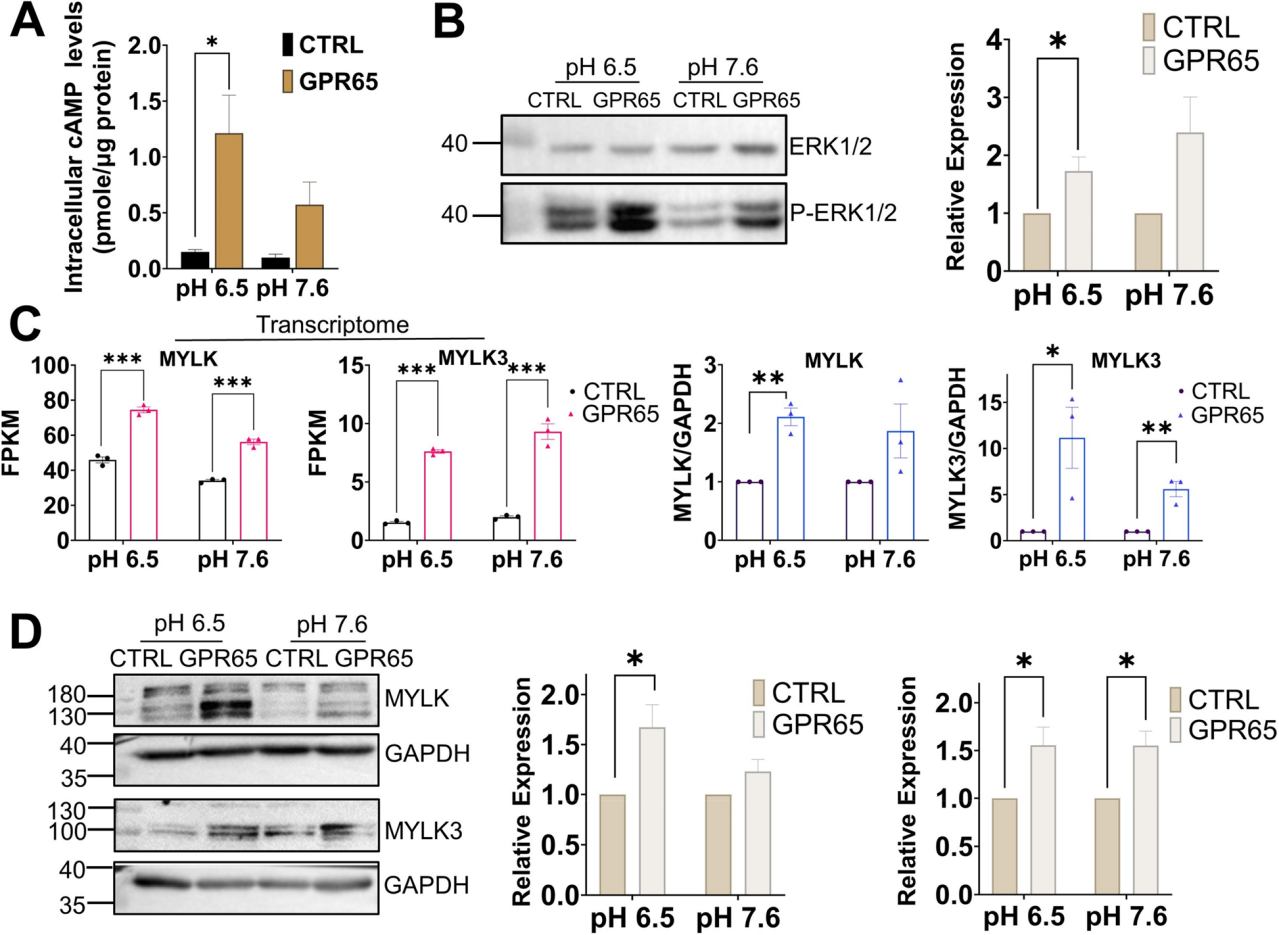
GPR65 upregulates MYLK and MYLK3 expression and remodels the cytoskeleton via cAMP-ERK. **A** GPR65-mediated cAMP production in cells under pH 6.5 conditions. **B** Detection of GPR65-mediated ERK phosphorylation (P-ERK) via western blotting. Cells were treated with serum-free medium at pH 6.5 or 7.6 for 24 h. **C** Transcriptome and RT‒qPCR analysis of MYLK and MYLK3 mRNA levels in GPR65-overexpressing HTR-8/SVneo cells. **D** Western blotting of MYLK and MYLK3 in HTR-8/SVneo cells treated with serum-free medium at pH 6.5 or 7.6 for 24 h. Results are presented as the mean ± SEM of at least three independent experiments, and the statistical analysis is shown: *P < 0.05, **P < 0.01, and ***P < 0.001

The activation of cAMP by GPR65 was confirmed using ELISA (Fig. 5A). To confirm whether downstream signals of the cAMP pathway were activated, we detected the phosphorylation of ERK protein. The results indicated that GPR65 activated the phosphorylation of ERK in HTR-8/SVneo cells at both pH 6.5 and pH 7.6 (Fig. 5B). According to the transcriptome results showing that GPR65 significantly changed the cytoskeleton, we detected the genes MYLK and MYLK3, which are closely related to the cytoskeleton in the ERK downstream pathway. Transcriptome, qPCR, and WB results revealed that the expression levels of MYLK and MYLK3 were significantly increased in cells along with the upregulation of GPR65 (Fig. 5C, D).

**Fig. 6:**
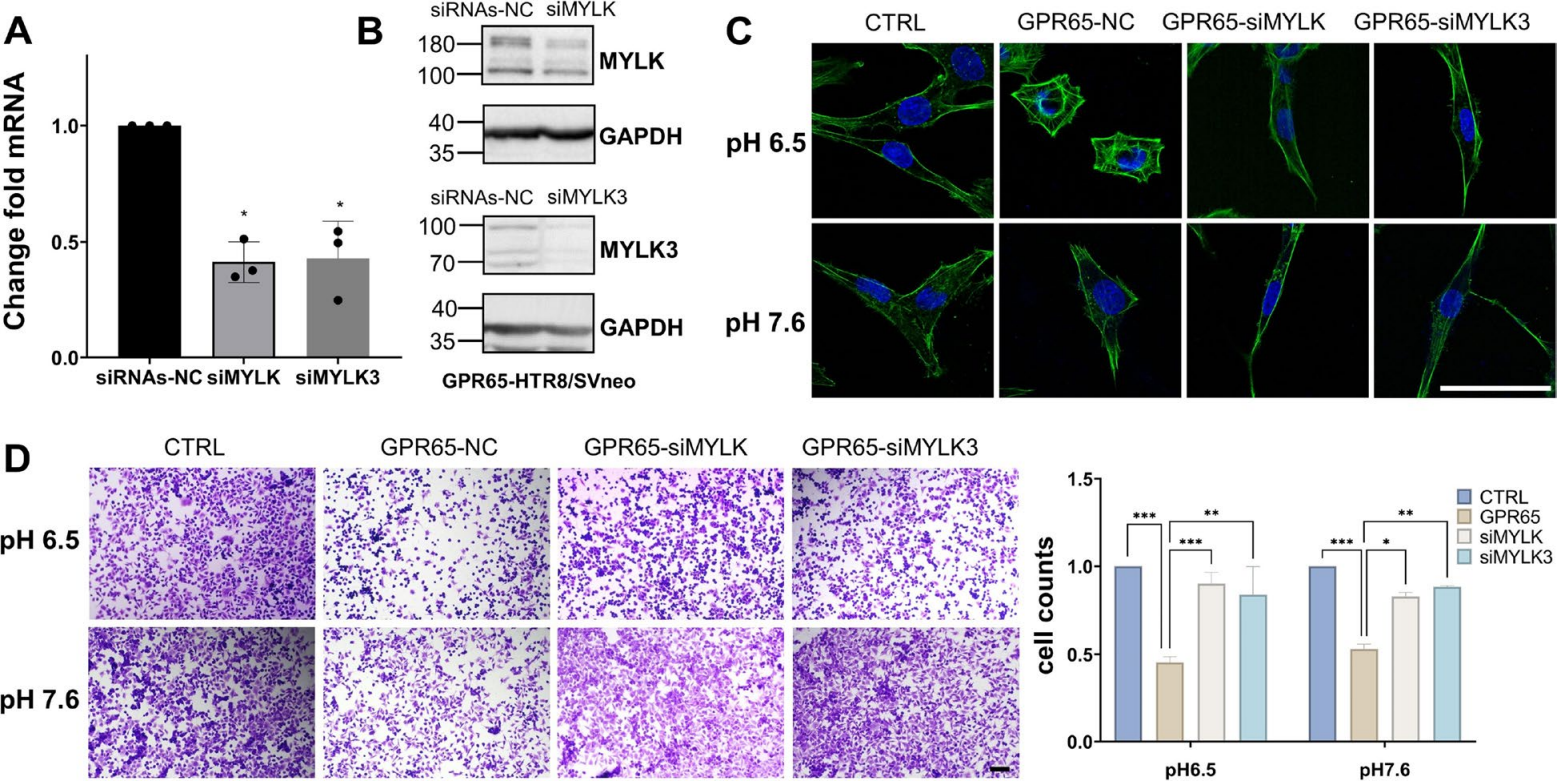
MYLK and MYLK3 knockdown rescues cytoskeletal changes and adhesion in GPR65-induced HTR-8/SVneo cells. **A**-**B** RT‒qPCR and western blotting were used to evaluate the knockdown efficiency of MYLK and MYLK3. **C** Actin organization was visualized by immunofluorescence after siMYLK and siMYLK3 transfection for 48 h. **D** The levels of cell invasiveness after siMYLK and siMYLK3 transfection for 48 h were examined by adhesion assay. Scale bars: 100 μm. Results are expressed as the mean ± SEM of at least three independent experiments, and the statistical analysis is shown: *P < 0.05, **P < 0.01, and ***P < 0.001

To further verify that MYLK and MYLK3 play a vital role in regulating cell motility by GPR65, siRNA was applied to silence MYLK and MYLK3 in GPR65-overexpressing HTR-8/SVneo cells. The qPCR and WB results indicated effective knockdown of MYLK and MYLK3 (Fig. 6A, B). Based on immunofluorescence detection of actin changes, knockdown of MYLK and MYLK3 increased linear actin filaments and reversed the shortening of actin filaments induced by GPR65 compared to GPR65-overexpressing HTR-8/SVneo cells (Fig. 6C). In addition, adhesion experiments suggested that the knockdown of MYLK and MYLK3 increases the number of adherent cells compared to GPR65-overexpressing HTR-8/SVneo cells, reversing the decrease in adhesion caused by GPR65 (Fig. 6D). These findings indicate that GPR65 regulates HTR-8/SVneo cell adhesion through the cAMP-ERK-MYLK axis at low pH environment.

### GPR65 inhibits HTR-8/SVneo cell adhesion by reducing fibronectin at low pH environment

**Fig. 7:**
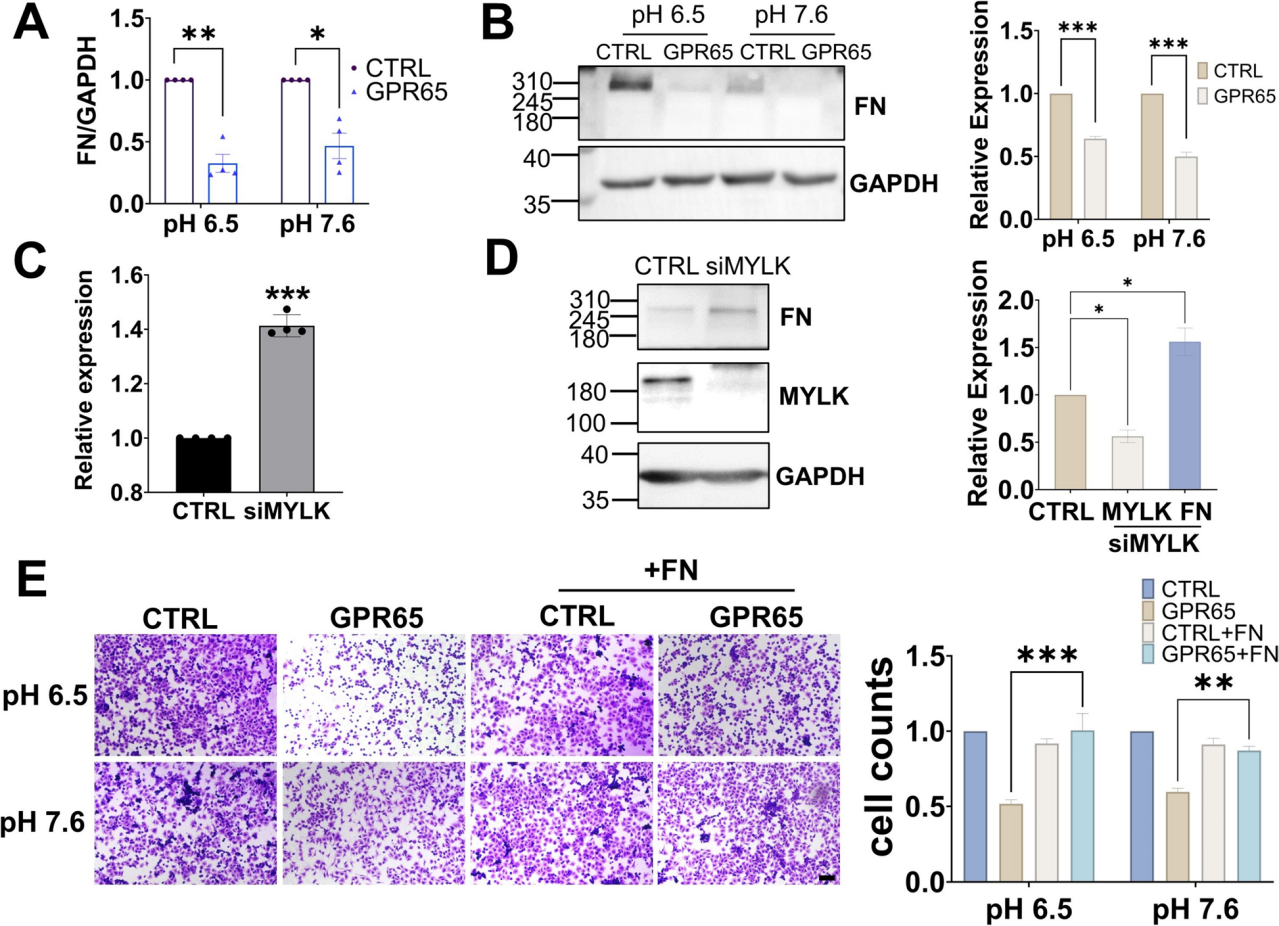
GPR65 inhibits the adhesion of HTR-8/SVneo cells by reducing FN. **A** RT‒qPCR analysis of FN mRNA levels in GPR65-overexpressing HTR-8/SVneo cells. **B** Western blotting of FN in HTR-8/SVneo cells treated with serum-free medium at pH 6.5 or 7.6 for 24 h. **C** RT‒qPCR analysis of FN mRNA levels in GPR65-overexpressing HTR-8/SVneo cells after siMYLK treatment for 48 h. **D** Western blotting of FN in GPR65-overexpressing HTR-8/SVneo cells after siMYLK treatment for 48 h. **E** Adhered HTR-8/SVneo cells were stained and counted after 2 h on FN (0 and 50 μg/mL)-coated 96-well plates. Scale bars: 100 μm. Results are presented as the mean ± SEM of at least three independent experiments, and the statistical analysis is shown: *P < 0.05, **P < 0.01, and ***P < 0.001

Transcriptome analysis revealed significant changes in ECM components, with numerous genes upregulated except for fibronectin (FN) downregulated (Fig. S7). FN is a major ECM that regulates cell adhesion by binding to integrins [26]. Therefore, it is speculated that GPR65 inhibits cell adhesion by downregulating the expression of FN. Furthermore, qPCR and WB assays demonstrated that FN was markedly downregulated upon overexpression of GPR65 (Fig. 7A, B). Interestingly, even though RT‒qPCR and WB detection showed that GPR65 upregulated the expression of integrin α5 (Fig. S8), it did not significantly impact cell adhesion. While integrin α5 has been identified as an ECM receptor [27], it is not a critical factor in this process.

To investigate the role of MYLK in this process, we examined the expression of FN in HTR-8/SVneo cells overexpressing GPR65 by siMYLK. The results revealed that the downregulation of MYLK resulted in a pronounced upregulation of FN expression levels (Fig. 7C, D). Thus, it was speculated that MYLK reverses the decrease in cell adhesion caused by GPR65 by upregulating FN. Furthermore, in vitro cell adhesion experiments with the addition of FN showed that exogenous FN could reverse the cell adhesion decline induced by GPR65 at pH 6.5 and 7.6 (Fig. 7E). Hence, GPR65 negatively regulates cell adhesion by the MYLK-FN pathway.

### Increased expression of GPR65 in villous tissue during early pregnancy loss

**Fig. 8:**
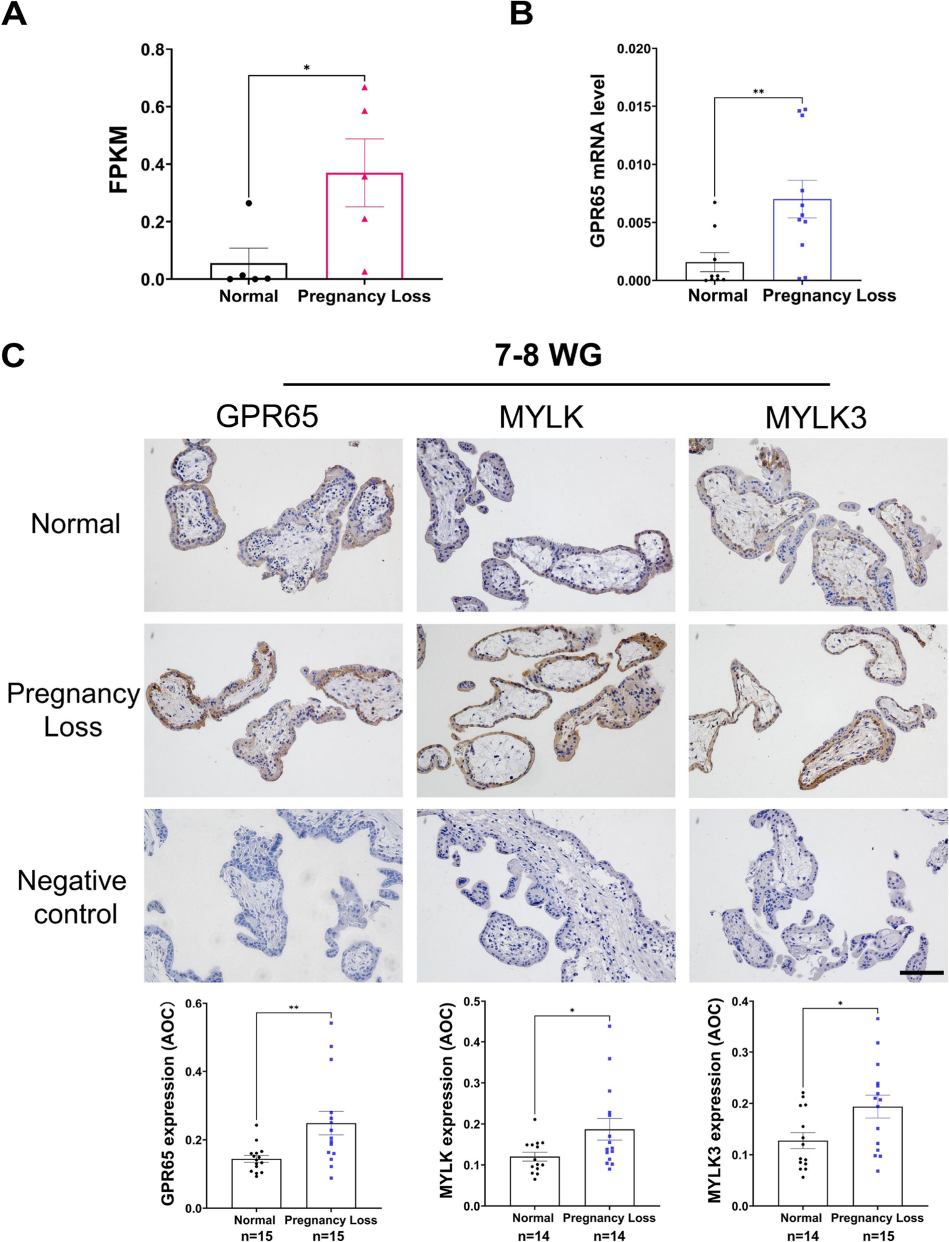
Increased expression of GPR65 in the villous tissue of pregnancy loss. **A** Transcriptome analysis of GPR65 expression in the villous tissue of pregnancy loss patients. **B** RT‒qPCR analysis of GPR65 in villous tissue of pregnancy loss patients (*n* = 7, *n* = 11). **C** Representative images of IHC and quantification results for GPR65, MYLK, and MYLK3 in control and pregnancy loss villous tissue. Scale bars: 100 μm. Results are presented as the mean ± SEM, and the statistical analysis is shown: *P < 0.05 and **P < 0.01

Due to the decreased adhesion function of trophoblast cells, the establishment of the maternal–fetal interface and subsequent placental development will be affected, leading to pregnancy loss or fetal growth retardation and other adverse pregnancy outcomes [28]. Accordingly, we analyzed the villi transcriptome data of PRJNA658420 in NCBI and found that GPR65 was highly expressed in the villous tissue of pregnancy loss (Fig. 8A). Similarly, the clustering heatmap based on differential gene expression levels showed that GPR65 was highly expressed in villous tissue (Fig. S9). Moreover, qPCR results confirmed that GPR65 expression levels were augmented in villous tissue from cases of pregnancy loss compared to normal samples (Fig. 8B). Additionally, IHC results also showed that GPR65, MYLK and MYLK3 protein levels were prominently increased in villous tissue in pregnancy loss (Fig. 8C). However, MYLK and MYLK3 were not upregulated in the villous tissue of pregnancy loss in qPCR results (Fig. S10), which may be attributed to their low expression level as well as the presence of multiple cell lines in the villous tissue. These results suggest that GPR65 and its downstream signaling pathways are associated with pregnancy loss.

## Discussion

**Fig. 9:**
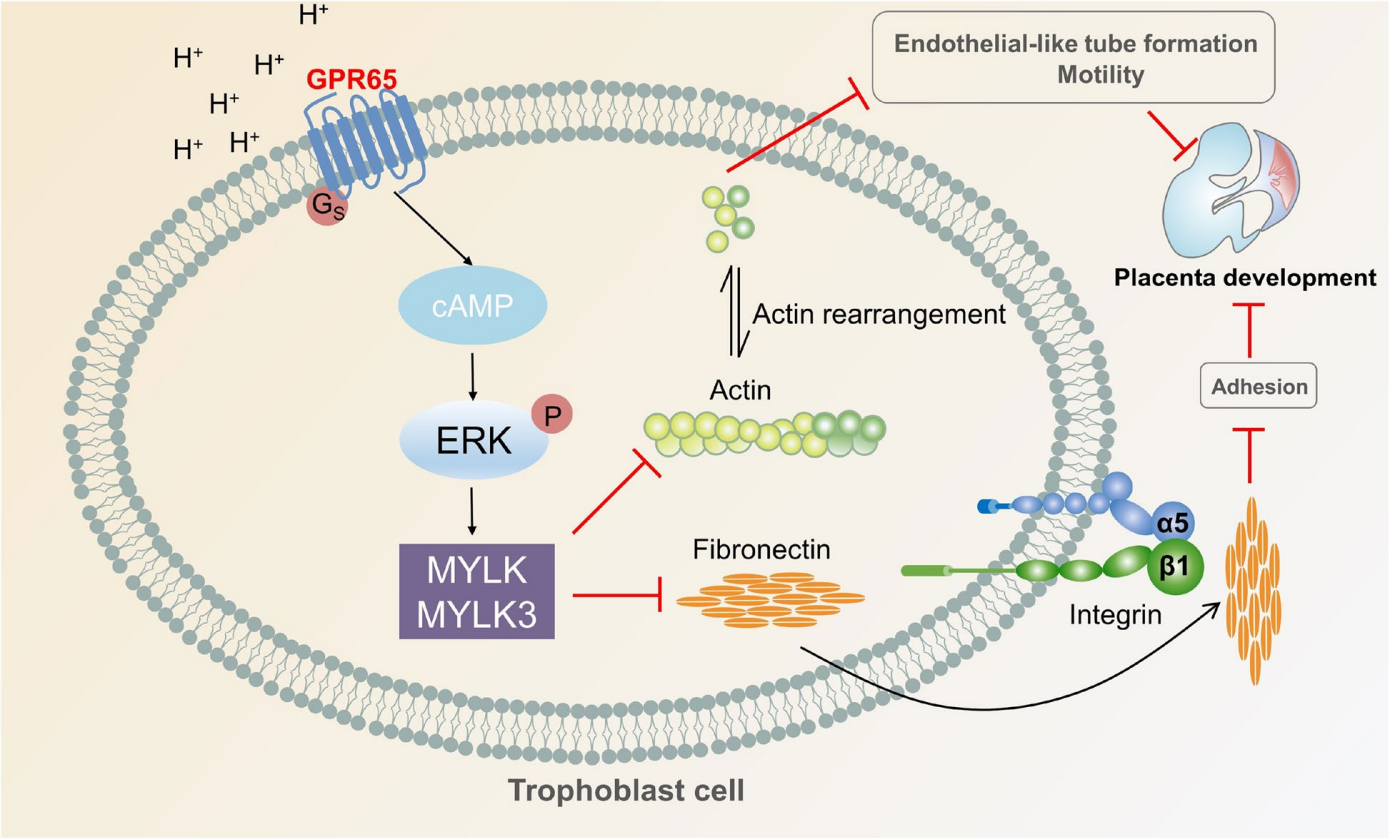
A schematic illustration of GPR65-regulated trophoblast cells. GPR65 reduces the adhesion of trophoblast cells by targeting MYLK and FN, which changes the actin cytoskeleton and inhibits endothelial-like tube formation

During the first trimester, the junction of the maternal arteries with the placental intervillous space is aggregated by EVTs, forming EVT plugs that block maternal blood flow to the placenta, which creates a severely hypoxic microenvironment that persists for up to 10 weeks of gestation [29]. Extracellular H^+^ ion accumulation caused by hypoxia reduces the pH of the surrounding environment [30]. While a low pH environment can promote trophoblast invasion and differentiation, it also presents a risk of acidosis due to the accumulation of carbonic and lactic acids in the fetus [31]. However, the mechanisms that regulate the extracellular acidic environment by trophoblast cells remain unclear. Our findings propose that GPR65 hinders the adhesion, migration, invasion, and endothelial-like tube formation of HTR8/SVneo cells, playing a negative role in placental development. Furthermore, in a low pH environment, GPR65 acts as an acid sensor to increase MYLK and MYLK3 expression through the cAMP-ERK signaling pathway and inhibit FN expression, thereby disrupting cytoskeleton remodeling, inhibiting cell adhesion, and impairing endothelial-like tube formation (Fig. 9).

During implantation, the pH of the endometrium decreases [32]. Although the exact pH value outside the trophoblast cells during early placenta formation is unknown, multiple lines of evidence suggest that blastocysts develop in a low-pH environment in early pregnancy [33]. The findings from the JAR spheroids and mouse blastocyst models suggest that GPR65 plays an inhibitory role during embryo implantation by affecting cell adhesion, growth, and invasion. As the placenta develops and maternal–fetal blood circulation is established, oxygen levels increase, and extracellular pH rises [34, 35]. Our study found that increasing the extracellular pH can alleviate the abnormal endothelial-like tube formation caused by GPR65 (Fig. 3C). Moreover, GPR65 did not inhibit the growth of JAR spheroids at pH 7.6, suggesting that growth inhibition could be rescued at high pH environment. These results suggest that GPR65 exerts a more severe inhibitory effect on cellular functions at low pH environment.

To further understand the role of GPR65 in trophoblast cell function, we conducted transcriptome analysis on HTR-8/SVneo cells under low pH conditions. The results showed that GPR65 regulates the actin cytoskeleton and ECM receptors. The actin cytoskeleton plays a crucial role in maintaining cell shape, motility, and intracellular transport [36]. Additionally, actin filaments can be remodeled to provide power for cell migration and invasion [37]. At low pH, GPR65 inhibits the formation of actin filaments in HTR-8/SVneo cells, consistent with its activation of RhoA signaling and actin rearrangement in CHO-S cells [12]. Microtubules are also significant for trophoblast cell motility and invasion [38, 39]. Stathmin is a microtubule-destabilizing protein, and its hyperphosphorylation can lead to increased microtubule stability and inhibition of trophoblast motility [40]. An endothelial-like tube formation assay revealed that GPR65 could impede the endothelial-like tube formation of cells in a low pH environment by inhibiting cytoskeleton formation and microtubule polymerization, thus reducing angiogenic ability. Cytoskeletal dynamics impact vascular growth, remodeling of endothelial cells, and regulating intravascular trophoblasts that are relevant to uterine spiral artery remodeling [41, 42]. Therefore, these findings indicate that GPR65 plays a critical role in regulating placental trophoblast cell function and endothelial-like tube formation through its effect on the actin cytoskeleton and microtubule dynamics.

MYLK is a protein kinase that is dependent on Ca^2+^/CaM (calmodulin) and promotes cell contraction by regulating the phosphorylation of the myosin light chain (RLC) [43]. As a component of the actin cytoskeleton, MYLK is involved in cellular processes such as cell adhesion, migration, and regulation of mechanical properties [44]. In MCF10A breast epithelial cells, MYLK loss has been evidenced to increase their motility and invasiveness and to activate signals such as ERK, which may promote tumor metastasis [45]. In tumor studies on Lewis lung carcinoma cells, GPR65 has been proven to promote cell survival and growth by activating the cAMP-ERK signaling pathway [11]. We confirmed that overexpression of GPR65 upregulates MYLK and MYLK3 in HTR-8/SVneo cells through qPCR and WB. Further experiments involving cytoskeleton staining with phalloidin and adhesion assays using siRNA-mediated knockdown of MYLK and MYLK3 in GPR65-overexpressing cells demonstrated that these two molecules are crucial targets of GPR65 in regulating cell adhesion.

As previously mentioned, MYLK can act as a scaffold and integrate cytoskeletal structures. MYLK and integrin α5 were detected by FN pulldown from the total protein of mouse smooth muscle cell lysates, indicating the involvement of MYLK in FN-integrin-cytoskeletal linkages [46]. MYLK regulates cell motility by stabilizing the cytoskeleton rather than by RLC phosphorylation. FN is one of the most abundant glycoproteins in the ECM, linking it to the actin cytoskeleton through integrins [47]. FN is present in the EVTs, endovascular trophoblast cells, and distal columns, where it can promote the adhesion and migration of trophoblast cells to maternal tissues [48]. During implantation in human blastocysts, FN interacts with CD26/dipeptidyl peptidase IV (a marker molecule of the endometrium) to encourage blastocyst adhesion [49]. Insulin-like growth factor 1 enhances FN expression on blastocysts, promoting their attachment to Ishikawa and invasiveness [50]. Interestingly, a hypoxic environment can promote FN expression, but the mechanism is not yet fully understood [51]. FN is also involved in GPR65-induced cytoskeletal changes, where silencing MYLK can reverse the downregulation of FN in cells overexpressing GPR65. Additionally, the addition of FN in vitro can promote cell adhesion. Studies have shown that FN expression is downregulated in the chorionic villi of spontaneous abortion patients and spontaneous abortion mice [52]. This indicates that the adhesion inhibition of GPR65 in mouse blastocysts may be achieved by down-regulating FN. Further investigations are needed to elucidate the underlying molecular mechanisms by which GPR65 regulates FN expression and its impact on blastocyst adhesion.

Moreover, in GPR65-overexpressing HTR-8/SVneo cells, the upregulation of integrin α5 may be caused by the activation of inside-out signaling resulting from the upregulation of MYLK. Research has shown that MYLK phosphorylation induced by integrins can lead to platelet retraction via outside-in signaling [53]. Although the G protein subunit Ga13 can bind directly to integrin aIIbb3 to regulate cell contraction, it is unclear whether integrin can bind to the G protein subunit when GPR65 is activated [54]. It has been determined that integrin α5 has no significant role in the GPR65-mediated inhibition of cell adhesion. Further investigation is required to identify other factors involved in cell adhesion that GPR65 regulates.

An interesting question to investigate is the impact of acidic and hypoxic microenvironments on the expression of GPR65. Our results showed that short-term (6 h) stimulation at pH 6.5 or hypoxic environment induced GPR65 expression in HTR-8/SVneo cells (Fig. S11 and Fig. S12). It indicates that the abnormal high expression of GPR65 in the placenta of pregnancy loss could be attributed to the presence of acidic and hypoxic microenvironments, which serve as inducing factors for GPR65 expression. Furthermore, it is important to consider that during the first trimester, the placenta exists in a physiological hypoxic and acidic environment. In pregnancy disorders such as preeclampsia and fetal growth restriction, the placenta is subjected to prolonged pathological hypoxia [55]. Exploring the expression and activity of GPR65 in hypoxic and acidic conditions holds significant value in understanding the molecular mechanisms underlying pregnancy-related disorders. At the same time, the influence of GPR65 on its expression level and cell function in the combined effect of pH and hypoxic microenvironment should be considered, which will help to explore the regulatory mechanism of GPR65 under physiological conditions.

The pathogenesis of PE is closely related to impaired trophoblast invasion and incomplete spiral artery remodeling, and placental hypoxia is the core of its pathogenesis [56]. During the occurrence of PE, trophoblast cells need to sense and respond to the persistent acidic extracellular environment induced by hypoxia. Our results showed that GPR65 inhibited the invasion and endothelial-like tube formation of HTR-8/SVneo cells. It is speculated that abnormal upregulation of GPR65 leads to insufficient remodeling of spiral arteries in trophoblast cells, which leads to the occurrence and development of PE. Uterine and placental dysfunction caused by preeclampsia may develop clinically into fetal growth restriction [57]. Therefore, further investigation into the molecular mechanisms by which GPR65 regulates trophoblast cells under hypoxic conditions will contribute to a better understanding of the pathogenesis of these diseases and may provide novel insights for the treatment of preeclampsia and fetal growth restriction.

A limitation of this study is the lack of understanding regarding the function and mechanisms of GPR65 in an animal model. While we employed JAR cell spheroids and mouse blastocysts as in vitro models to simulate embryo implantation, the investigation of GPR65 in transgenic animal models warrants further exploration. The use of animal disease models combined with clinical samples will better explain the pathological mechanisms of pregnancy diseases such as preeclampsia and fetal growth restriction, and provide new targets and research directions for the prevention and treatment of pregnancy diseases caused by pathological hypoxia.

## Conclusions

Overall, GPR65 affects trophoblast cell adhesion, migration, invasion and endothelial-like tube formation. Moreover, GPR65 regulates cytoskeleton remodeling and cell adhesion during this process by activating the cAMP-ERK signaling pathway to upregulate MYLK and MYLK3 and downregulate FN in low pH environment. By comparing GPR65 expression in pregnancy loss and normal villous tissues, it was found that the transcript and protein levels of GPR65 and the expression levels of MYLK and MYLK3 were significantly upregulated, suggesting that GPR65 may play a crucial role in early pregnancy loss. In addition, impaired trophoblast cell invasion can result in various pregnancy complications, such as miscarriage, preeclampsia, and fetal growth restriction [58–60]. Indeed, these diseases are known to be associated with pathological hypoxia and alterations in the acidic microenvironment. In-depth studies on GPR65 can offer new insights into the molecular mechanisms underlying placental development and the pathogenesis of these diseases.

### Supplementary Information

**Additional file 1: Fig. S1.** Construction of JAR cell spheroids overexpressing GPR65 and mouse blastocysts overexpressing *Gpr65*. **Fig. S2.** The expression level of GPR65 detected in GPR65-overexpressing or GPR65-silenced HTR-8/SVneo cells. **Fig. S3.** The effect of GPR65 on the adhesion of HTR-8/SVneo cells at different pH. **Fig. S4.** Effect of GPR65 on wound closure in HTR-8/SVneo cells at pH 6.5 and 7.6. **Fig. S5.** Effect of GPR65 on the proliferation of HTR-8/SVneo cells. **Fig. S6.** Transcriptome analysis of the RNA-sequence data for GPR65-overexpressing HTR-8/SVneo cells. **Fig. S7.** DEGs in GPR65-overexpressing HTR-8/Svneo cells showing significantly differentially expressed ECM genes. **Fig. S8.** GPR65 inhibits the expression of integrin α5 in HTR-8/Svneo cells. **Fig. S9.** Transcriptome analysis of human villi tissue from pregnant women diagnosed with embryo development cessation. **Fig. S10.** The expression of MYLK and MYLK3 in villous tissue of pregnancy loss. **Fig. S11.** Short-term induction of GPR65 expression under low pH conditions. **Fig. S12.** Induction of GPR65 expression under hypoxic conditions. **Table S1.** Clinical characteristics of the pregnant women enrolled in this study. **Table S2.** siRNA target sequences. **Table S3.** Primer sequences for RT‒qPCR.

**Additional file 2. **

## Data Availability

The data that support the findings of this study are available from the corresponding author upon reasonable request.
