# Correction: GPR65 inhibits human trophoblast cell adhesion through upregulation of MYLK and downregulation of fibronectin via cAMP-ERK signaling in a low pH environment

**DOI:** 10.1186/s12964-024-01801-9

**Published:** 2024-08-27

**Authors:** Jia Mao, Ying Feng, Yayun Zheng, Yaqiu Gao, Linyu Zhang, Xinrui Sun, Yilun Wu, Xiaofeng Zhu, Fang Ma

**Affiliations:** 1 Key Laboratory of Bio-Resource and Eco-Environment of Ministry of Education, College of Life Sciences, Sichuan University, Chengdu, 610064 Sichuan China; 2 Center for Translational Medicine, Key Laboratory of Birth Defects and Related Diseases of Women and Children (Sichuan University), Ministry of Education, West China Second University Hospital, Sichuan University, Chengdu, 610041 Sichuan China; 3 Department of Histology, Embryology and Neurobiology, West China School of Basic Medical Sciences & Forensic Medicine, Sichuan University, Chengdu, 610041 Sichuan China; 4 Department of Obstetrics and Gynecology, West China Second University Hospital, Sichuan University, Chengdu, 610041 Sichuan China

**Correction: Cell Communication and Signaling (2023) 21:238**

10.1186/s12964-023-01249-3

Following publication of the original article, the authors flagged the following error in the Results subsection, ‘GPR65 regulates HTR-8/SVneo cell adhesion via the cAMP-ERK‑MYLK axis in a low pH environment’: where it says “Transcriptome, qPCR, and WB results revealed that the expression levels of MYLK and MYLK3 were significantly increased in cells along with the upregulation of GPR65 (Fig. 5C, D)”, it previously said “Transcriptome, qPCR, and WB results revealed that the expression levels of MYLK and MYLK3 were significantly decreased in cells along with the upregulation of GPR65 (Fig. 5C, D)”. The article has since been updated to correct this error. The authors thank you for reading this erratum and apologize for any inconvenience caused.

