# Supplementary material for: GPR65 inhibits human trophoblast cell adhesion through upregulation of MYLK and downregulation of fibronectin via cAMP-ERK signaling in a low pH environment
Source: Cell Commun Signal. 2023 Sep 18;21:238. doi: 10.1186/s12964-023-01249-3 (PMC10506227; doi:10.1186/s12964-023-01249-3)
Supplement: Supplementary file 2 — Additional file 1: Fig. S1. Construction of JAR cell spheroids overexpressing GPR65 and mouse blastocysts overexpressing Gpr65. Fig. S2. The expression level of GPR65 detected in GPR65-overexpressing or GPR65-silenced HTR-8/SVneo cells. Fig. S3. The effect of GPR65 on the adhesion of HTR-8/SVneo cells at different pH. Fig. S4. Effect of GPR65 on wound closure in HTR-8/SVneo cells at pH 6.5 and 7.6. Fig. S5. Effect of GPR65 on the proliferation of HTR-8/SVneo cells. Fig. S6. Transcriptome analysis of the RNA-sequence data for GPR65-overexpressing HTR-8/SVneo cells. Fig. S7. DEGs in GPR65-overexpressing HTR-8/Svneo cells showing significantly differentially expressed ECM genes. Fig. S8. GPR65 inhibits the expression of integrin α5 in HTR-8/Svneo cells. Fig. S9. Transcriptome analysis of human villi tissue from pregnant women diagnosed with embryo development cessation. Fig. S10. The expression of MYLK and MYLK3 in villous tissue of pregnancy loss. Fig. S11. Short-term induction of GPR65 expression under low pH conditions. Fig. S12. Induction of GPR65 expression under hypoxic conditions. Table S1. Clinical characteristics of the pregnant women enrolled in this study. Table S2. siRNA target sequences. Table S3. Primer sequences for RT‒qPCR. [file 12964_2023_1249_MOESM1_ESM.docx]

**Supplementary Figures and Tables for**

**GPR65 inhibits human trophoblast cell adhesion through upregulation of MYLK and downregulation of fibronectin via cAMP-ERK signaling in a low pH environment**

Jia Mao^1,2#^, Ying Feng^3#^, Yayun Zheng^3^, Yaqiu Gao^1^, Linyu Zhang^2,4^, Xinrui Sun^2,4^, Yilun Wu^2,4^, Xiaofeng Zhu^1^*, Fang Ma^2,4^*

*^1^Key Laboratory of Bio-Resource and Eco-Environment of Ministry of Education, College of Life Sciences, Sichuan University, Chengdu, Sichuan 610064, China*

*^2^Center for Translational Medicine, Key Laboratory of Birth Defects and Related Diseases of Women and Children (Sichuan University), Ministry of Education, West China Second University Hospital, Sichuan University, Chengdu, Sichuan 610041, China*

*^3^Department of Histology, Embryology and Neurobiology, West China School of Basic Medical Sciences & Forensic Medicine, Sichuan University, Chengdu, Sichuan 610041, China*

*^4^Department of Obstetrics and Gynecology, West China Second University Hospital, Sichuan University, Chengdu, Sichuan 610041, China*

#These authors contributed equally to this work.

*Corresponding authors. E-mail address: zhuxiaofeng@scu.edu.cn; mafangmed@scu.edu.cn





Fig. S1. Construction of JAR cell spheroids overexpressing GPR65 and mouse blastocysts overexpressing *Gpr65*. (A) RT‒qPCR was used to evaluate the overexpression efficiency of GPR65 in JAR cells. (B) The expression level of GPR65 was detected by flow cytometry in GPR65-overexpressing JAR cells. (C) The cell morphology of JAR spheroids. (D) The cell morphology of JAR spheroids adhered to Ishikawa cells. (E) The morphology of mouse blastocysts. (F) The effect of *Gpr65*-GFP transfected mouse blastocysts detected by fluorescence microscope. Scale bars: 100 μm.


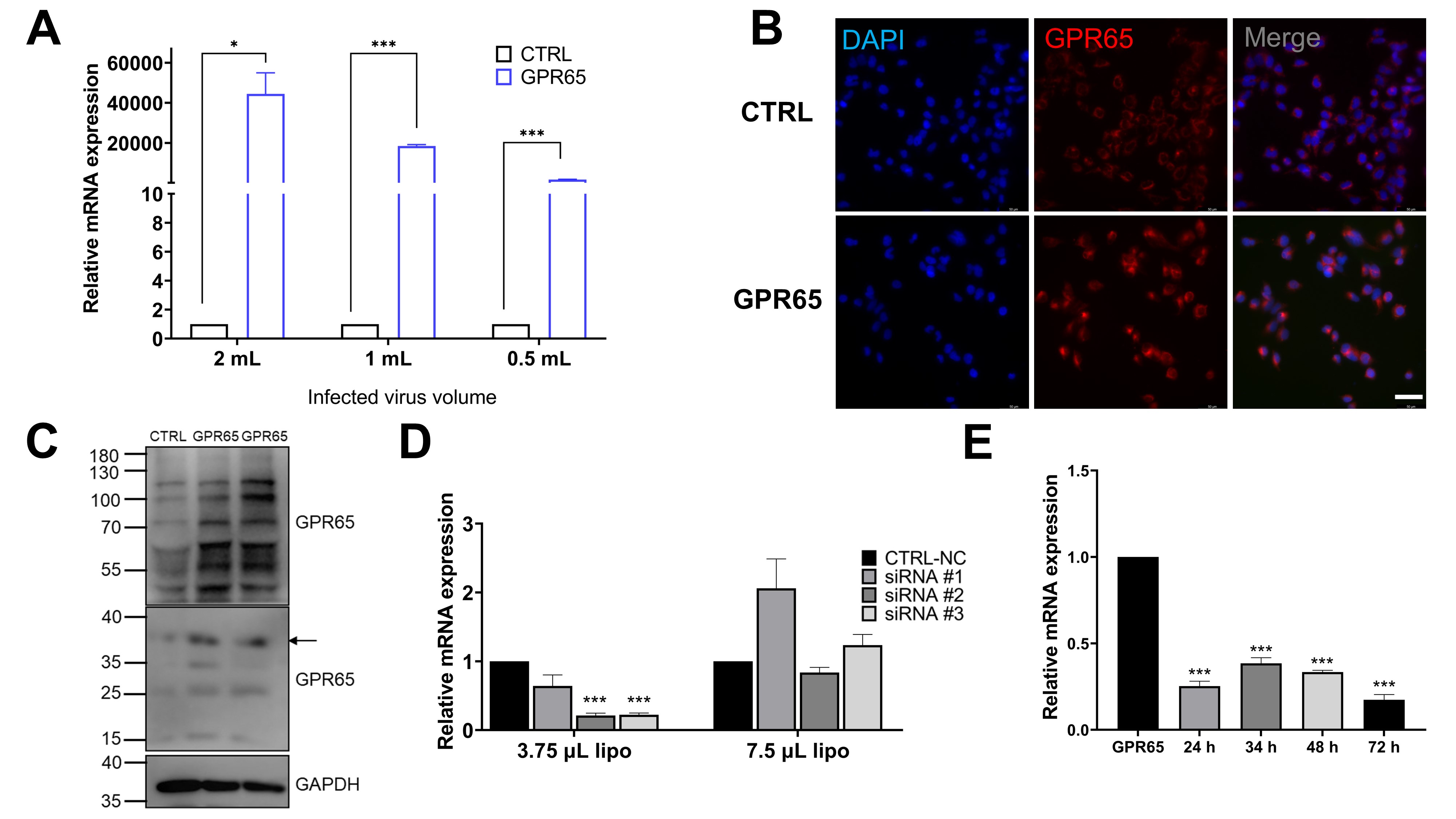


Fig. S2. The expression level of GPR65 detected in GPR65-overexpressing or GPR65-silenced HTR-8/SVneo cells. (A) The expression level of GPR65 detected by RT‒qPCR in HTR-8/SVneo cells infected with different virus volumes. (B) Immunofluorescence detection of the expression level of GPR65 in GPR65-overexpressing or GPR65-silenced HTR-8/SVneo cells. Scale bars: 50 μm. (C) Western blot detection of the expression level of GPR65 in HTR-8/SVneo cells. (D) The expression levels of GPR65 in HTR-8/SVneo cells silenced by different siRNAs and lipo3000 volume were detected by RT‒qPCR. (E) Detection of the silencing efficiency of siRNA in HTR-8/SVneo cells at different time points by RT‒qPCR.


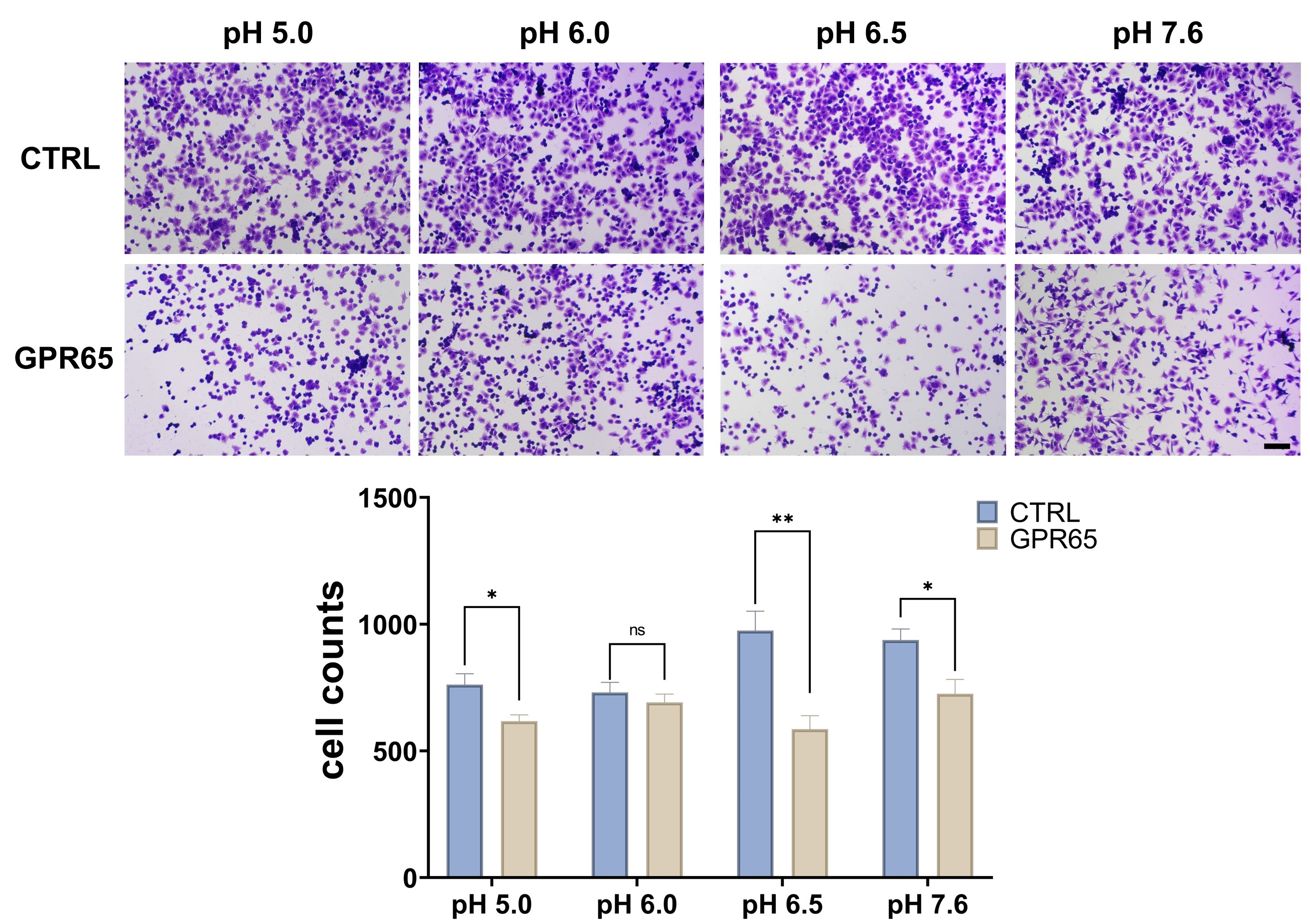


Fig. S3. The effect of GPR65 on the adhesion of HTR-8/SVneo cells at different pH. Scale bars: 100 μm. Results are expressed as the mean ± SEM of at least three images from three different views, and the statistical analysis is shown: *P< 0.05; **P <0.01.


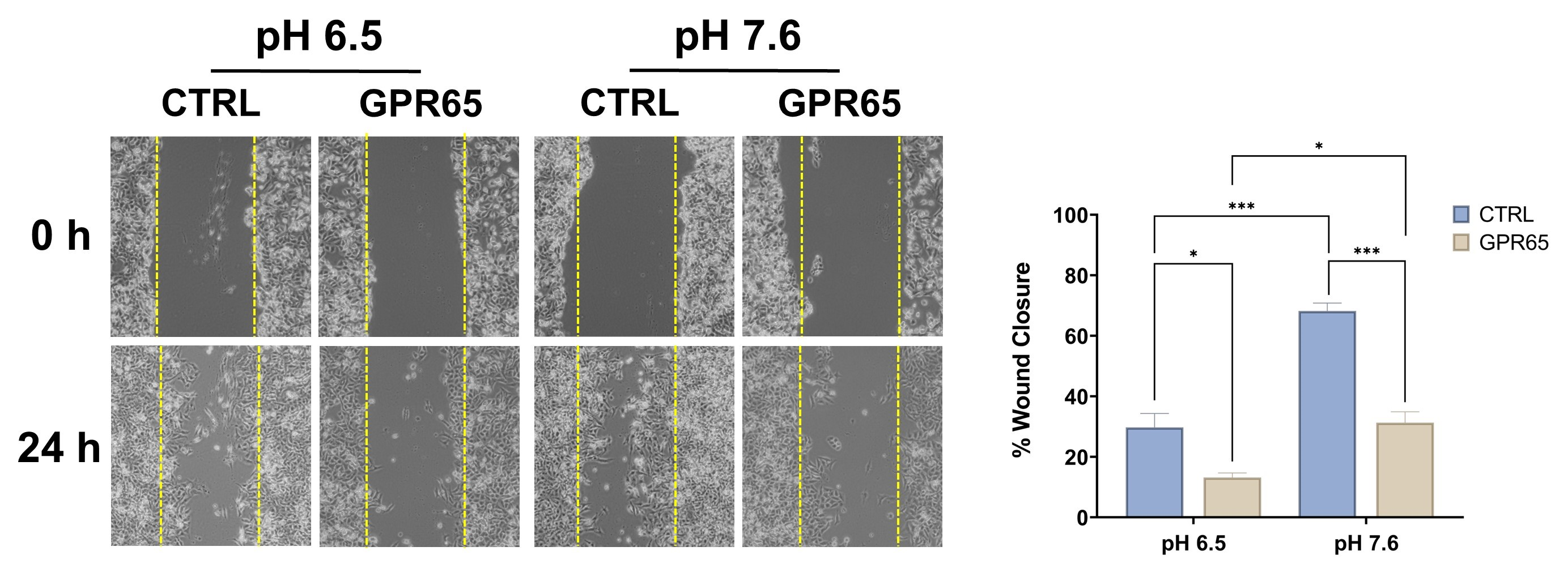


Fig. S4. Effect of GPR65 on wound closure in HTR-8/SVneo cells at pH 6.5 and 7.6.


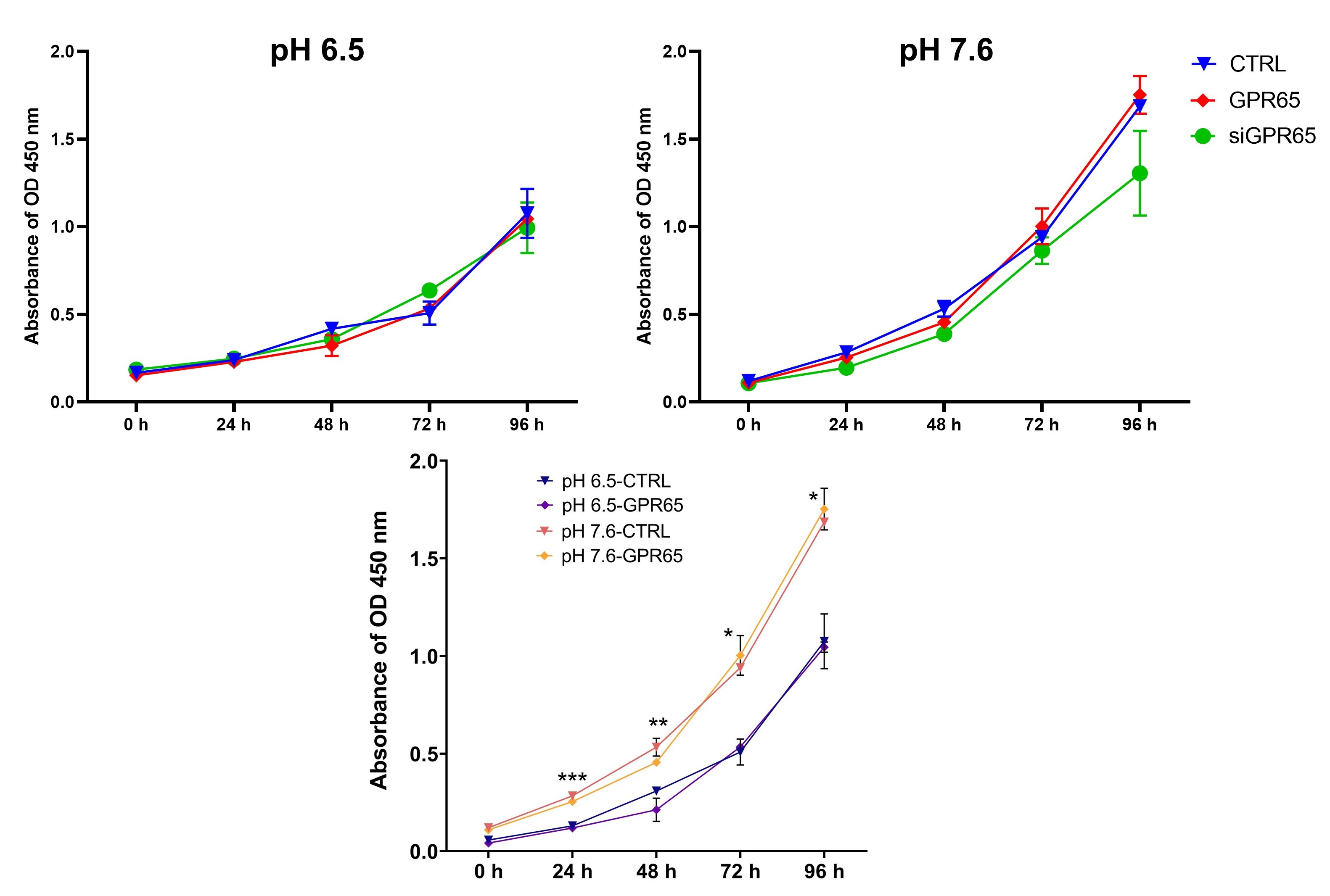


Fig. S5. Effect of GPR65 on the proliferation of HTR-8/SVneo cells. Cell proliferation was assessed using CCK8 assay under different pH conditions.


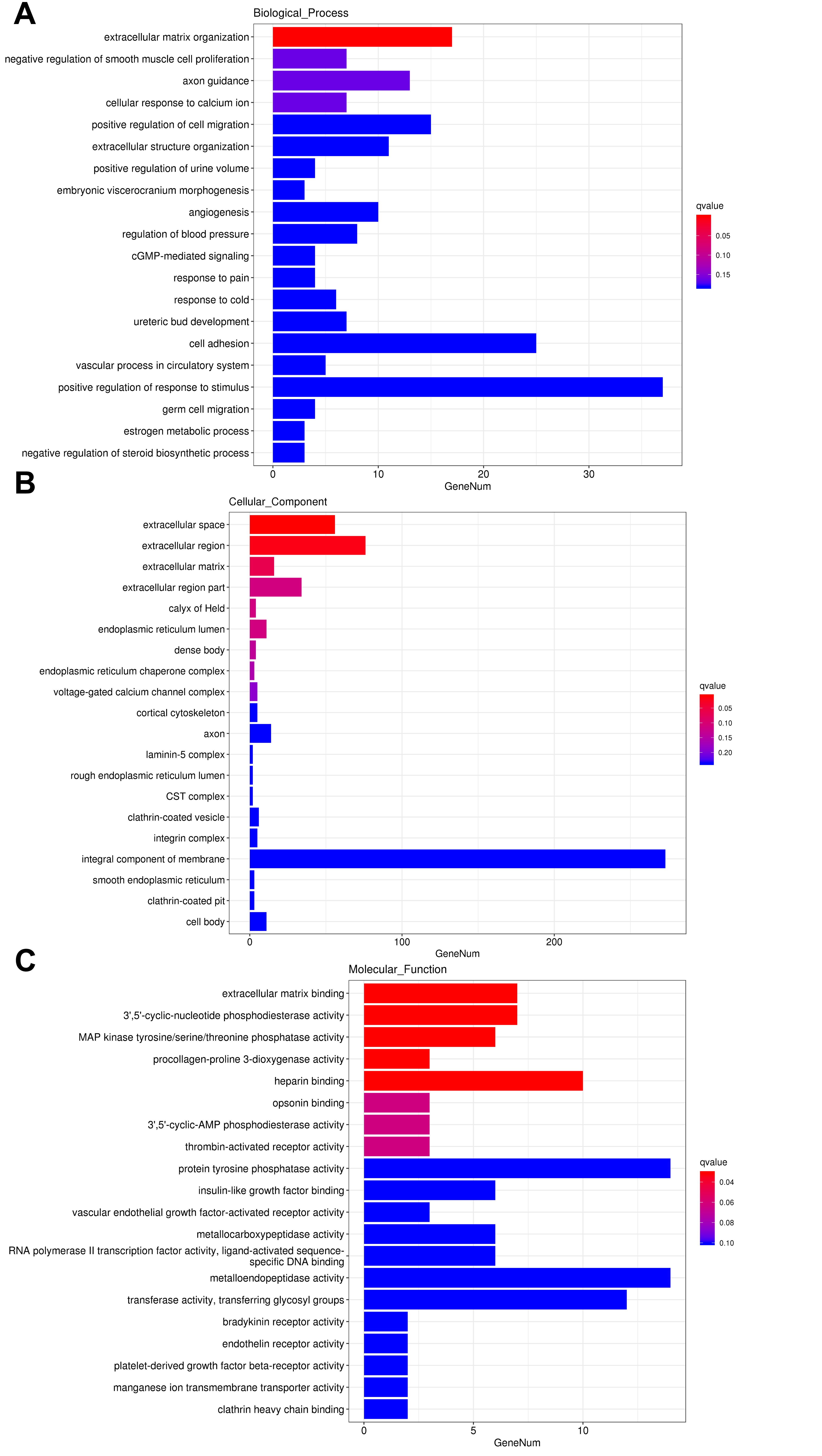


Fig. S6. Transcriptome analysis of the RNA-sequence data for GPR65-overexpressing HTR-8/SVneo cells. (A-C) The distribution of the DEGs in the GO categories is shown in biological process (BP), molecular function (MF), and cellular component (CC). The abscissa is GeneRatio, which is the ratio of the gene of interest annotated in this entry to all DEGs, and the ordinate is each GO annotation entry. The size of the point represents the number of differentially expressed genes annotated in the pathway, and the color of the point represents the q value of the hypergeometric test.


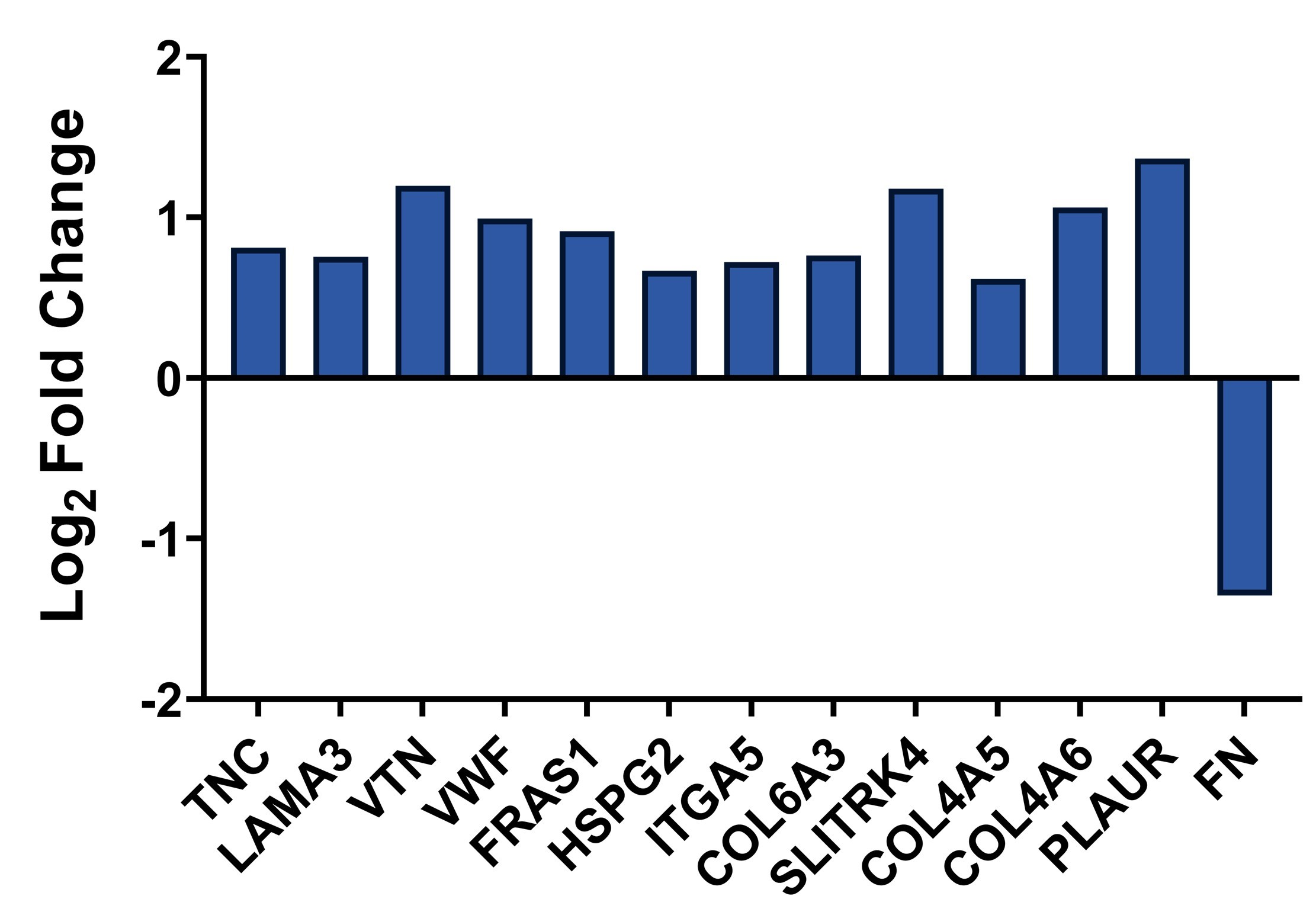


Fig. S7. DEGs in GPR65-overexpressing HTR-8/Svneo cells showing significantly differentially expressed ECM genes.


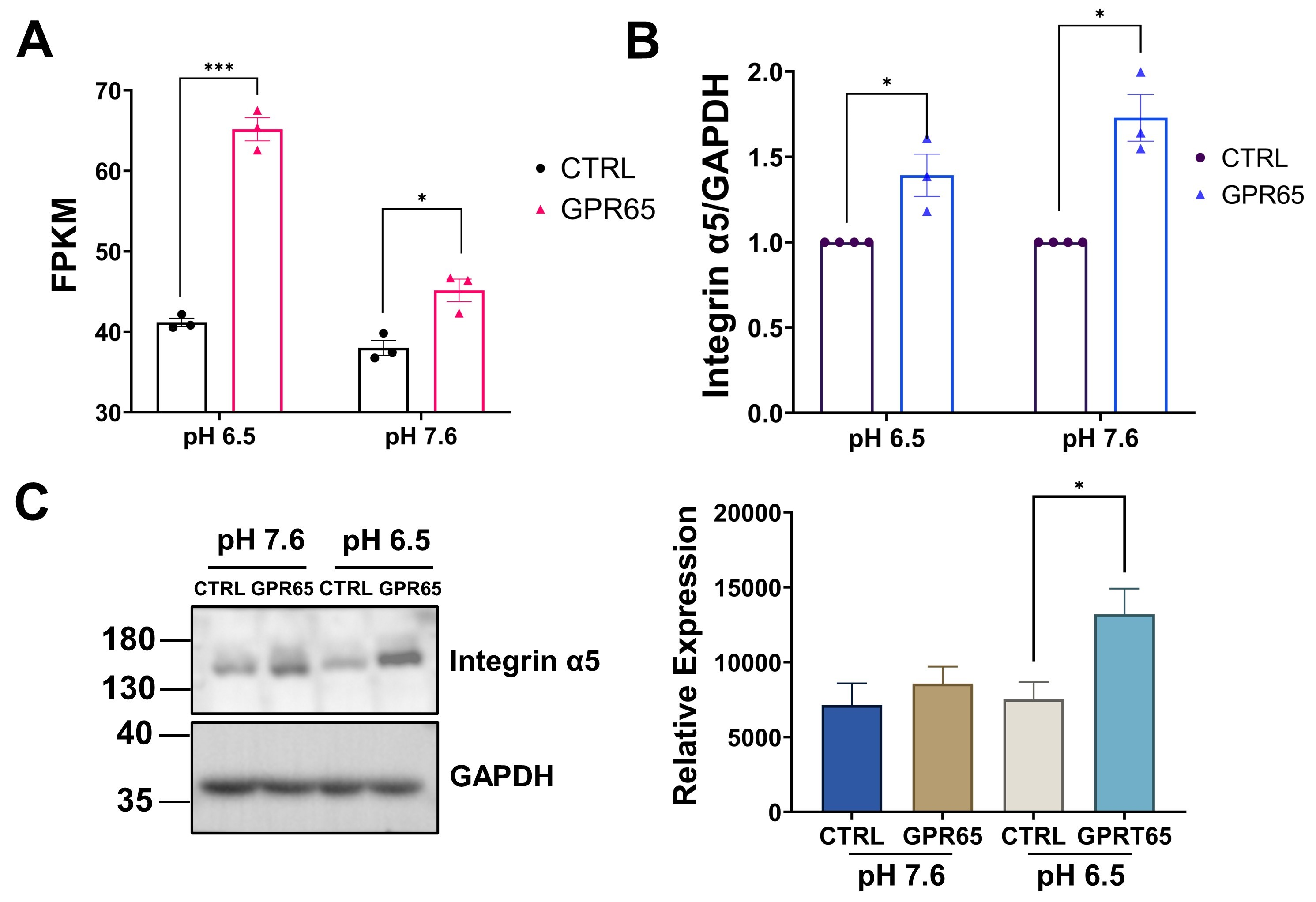


Fig. S8. GPR65 inhibits the expression of integrin α5 in HTR-8/Svneo cells. (A-B) Transcriptome and RT‒qPCR analysis of integrin α5 mRNA levels in GPR65-overexpressing HTR-8/SVneo cells. (C) Western blots of integrin α5 in HTR-8/SVneo cells treated with serum-free medium at pH 6.5 or 7.6 for 24 h. Results are expressed as the mean ± SEM of at least three independent experiments, and the statistical analysis is shown: *P < 0.05; **P <0.01; ***P < 0.001.


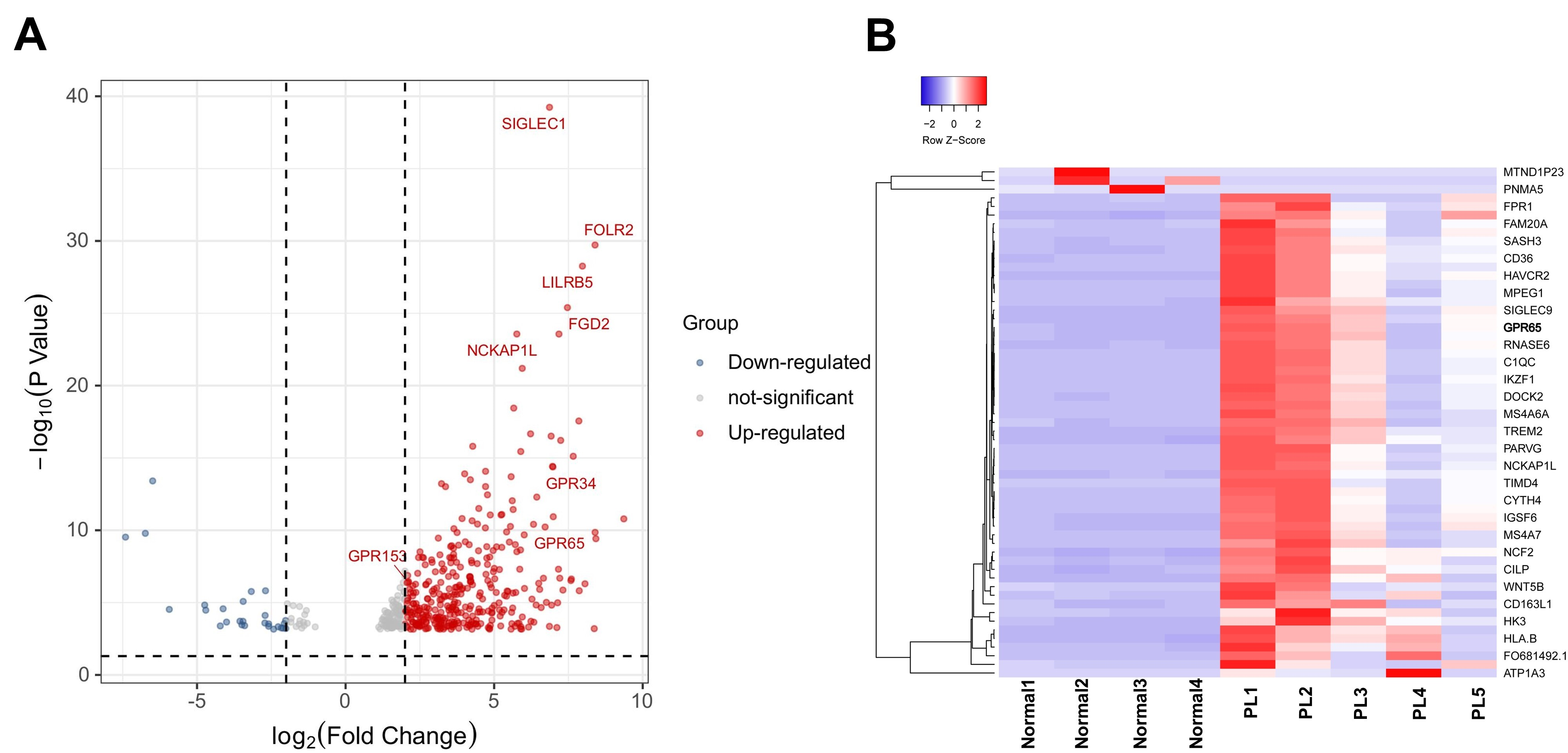


Fig. S9. Transcriptome analysis of human villi tissue from pregnant women diagnosed with embryo development cessation. (A) A volcano plot of RNA-seq data from villi of normal and pregnancy loss. The detection of DEGs with a fold change ≥ 2 and FDR < 0.05. (B) Cluster analysis of all the DEGs obtained from RNA-seq. PL: pregnancy loss.


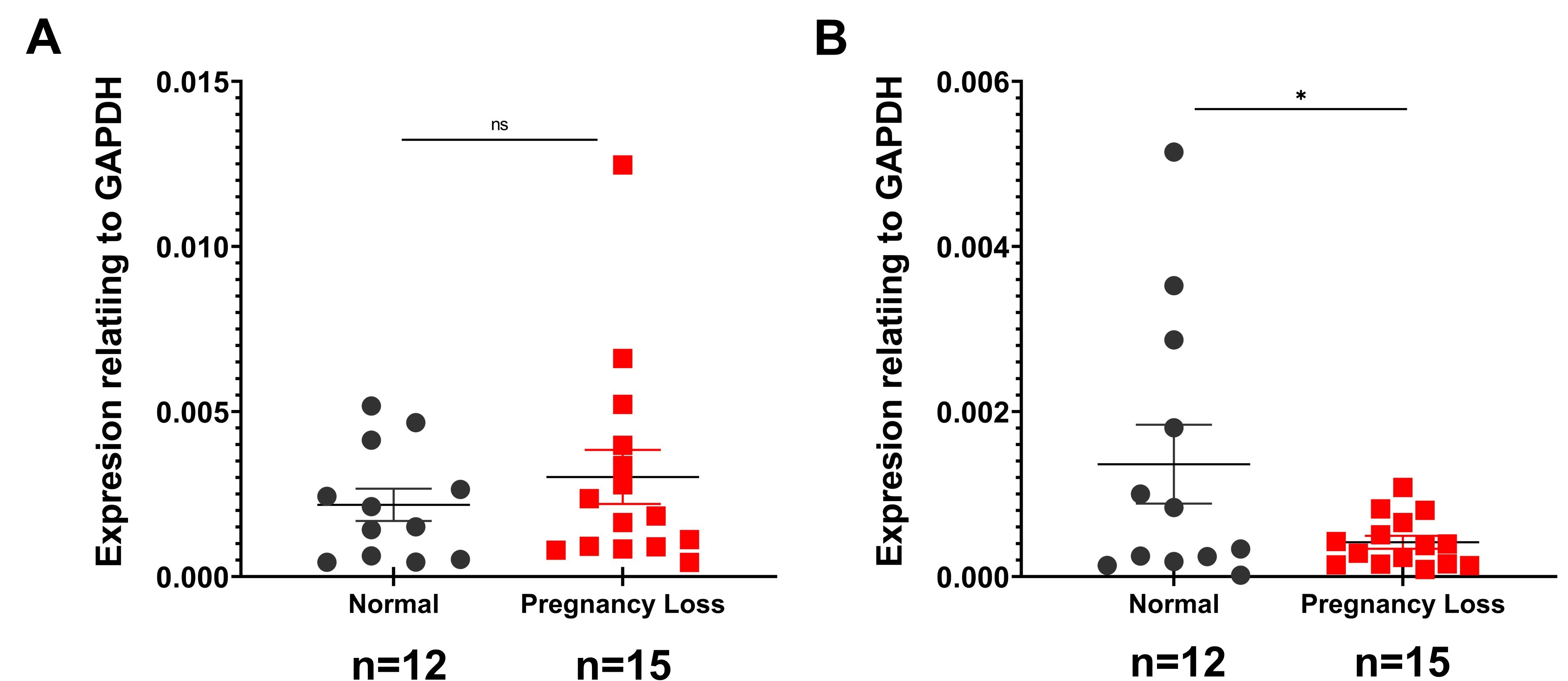


Fig. S10. The expression of MYLK and MYLK3 in villous tissue of pregnancy loss. (A, B) RT‒qPCR analysis of MYLK and MYLK3 in villous tissue of pregnancy loss. Results are expressed as the mean ± SEM, and the statistical analysis is shown: *P < 0.05.


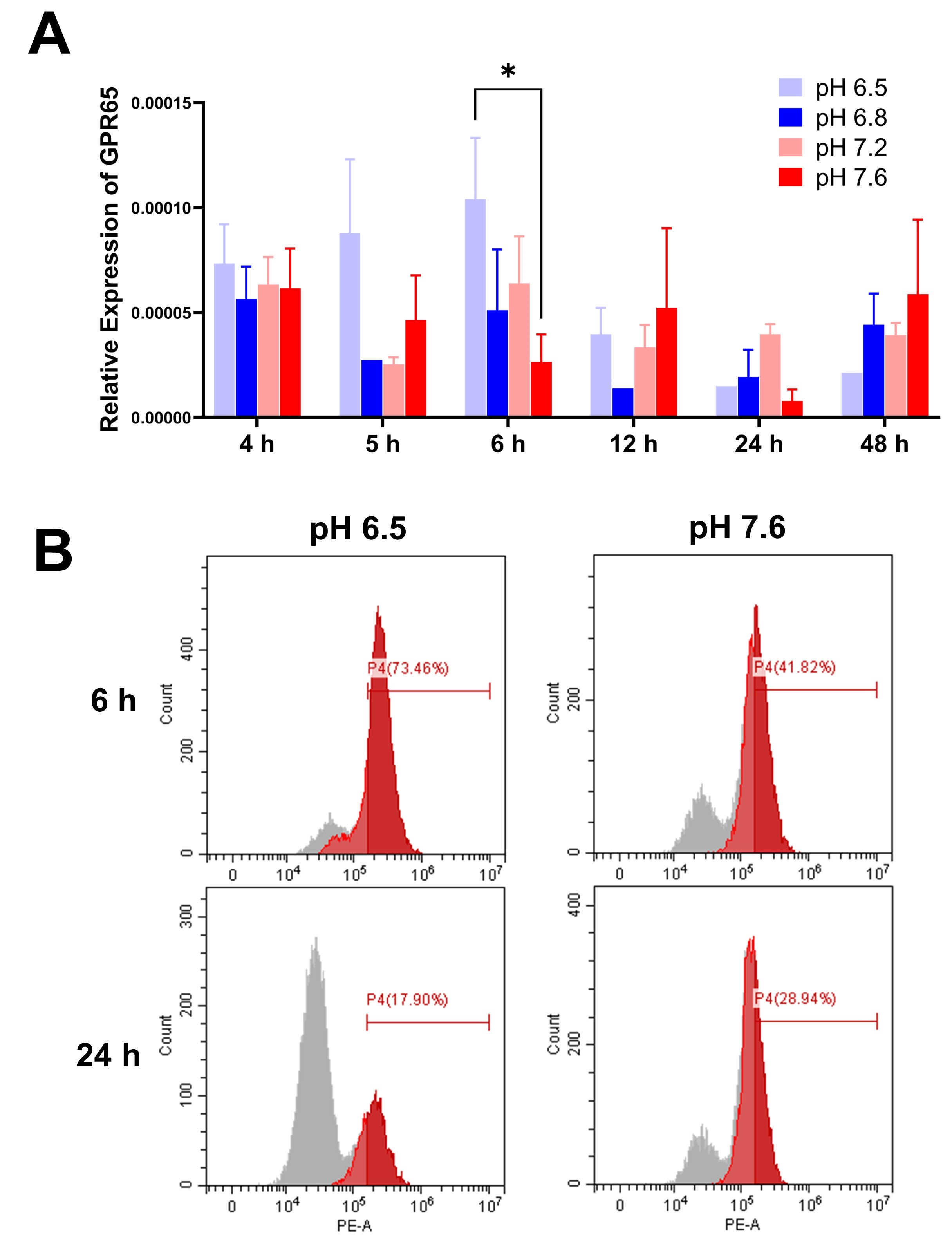
Fig. S11. Short-term induction of GPR65 expression under low pH conditions. (A) RT‒qPCR detection of the mRNA level of GPR65 after conditioned medium with different pH values. (B) The protein level of GPR65 was detected by flow cytometry in the conditioned medium at pH 6.5 and 7.6 for 6 h and 24 h.


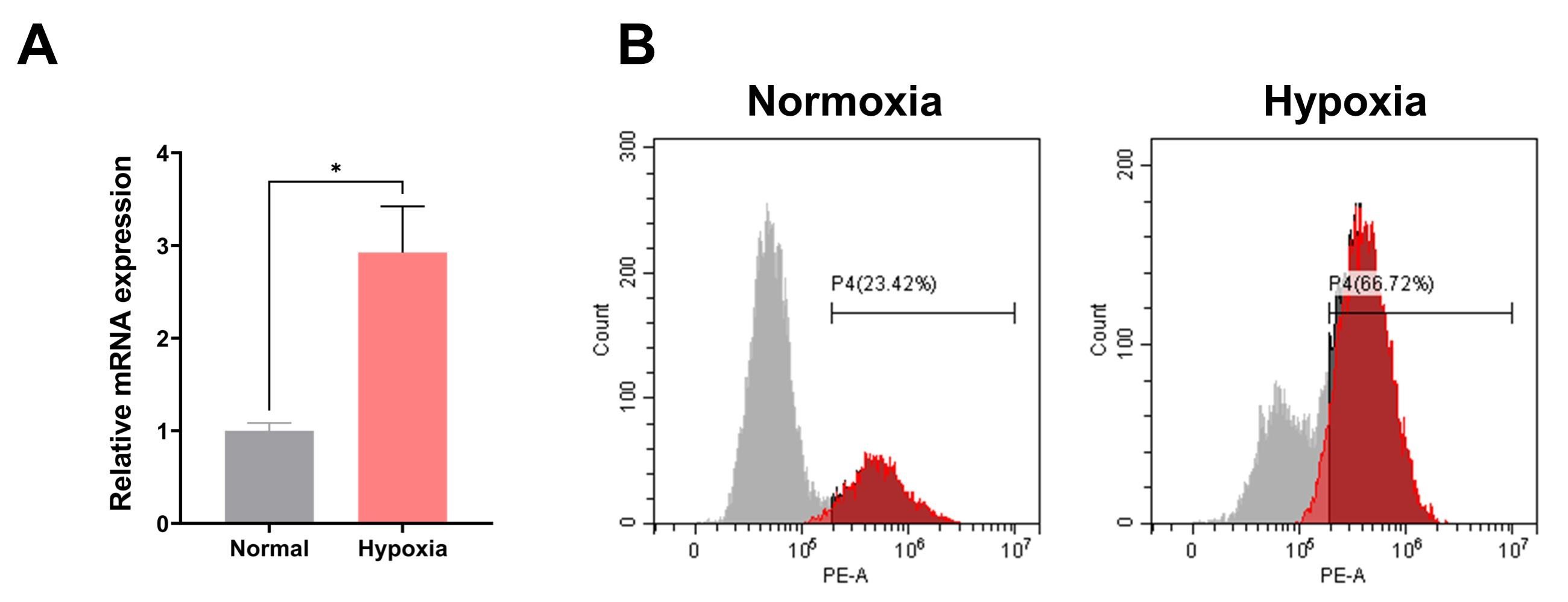


Fig. S12. Induction of GPR65 expression under hypoxic conditions. (A) RT‒qPCR detection of GPR65 mRNA levels under normoxic (20%) and hypoxic (3%) conditions. (B) The protein level of GPR65 was detected by flow cytometry under normoxic and hypoxic conditions.

**Table S1. Clinical characteristics of the pregnant women enrolled in this study**

| Characteristics | Normal  （n=15） | Pregnancy loss（n=15） | P value |
| --- | --- | --- | --- |
| Maternal age (y) | 28.67 ± 1.37 | 33.20 ± 1.24 | 0.021^*^ |
| Gravidity | 2.67 ± 0.43 | 2.45 ± 0.49 | 0.749 |
| Parity | 0.91 ± 0.26 | 0.64 ± 0.24 | 0.443 |
| Gestational Week | 7.00 ± 0.37 | 7.60 ± 0.45 | 0.306 |

Note: Values are expressed as the mean ± SEM, and statistical analyses were performed by using an independent-samples *t*-test in GraphPad Prism.

**Table S2. siRNA target sequences**

| Gene | Target sequence |
| --- | --- |
| GPR65 siRNA1 | TCACCTGCATTGCCGTTGA |
| GPR65 siRNA2 | CAACCGGAAAGTCTACCAA |
| GPR65 siRNA3 | GAATATTGCGATGCCGAAA |
| MYLK siRNA1 | GACGGGAACTGCTCTTTAA |
| MYLK siRNA2 | CTAAGACCATTCGCGATTT |
| MYLK siRNA3 | GCAAGGCTGTCAACAGTCT |
| MYLK3 siRNA1 | GCCAAAGCTTCAAGATCCA |
| MYLK3 siRNA2 | GCACCCTTGTCATGGAGTA |
| MYLK3 siRNA3 | GGATCACAGATGAGAAGTA |

**Table S3. Primer sequences for RT‒qPCR**

| Human Gene | Target sequence | |
| --- | --- | --- |
| GPR65 | F | GCATTGCCGTTGATCGGTATT |
|  | R | CGTCCTGAACAAGTTGAGGTT |
| MYLK | F | GAATATTGCGATGCCGAAA |
|  | R | GACGGGAACTGCTCTTTAA |
| MYLK3 | F | CTAAGACCATTCGCGATTT |
|  | R | GCAAGGCTGTCAACAGTCT |
| Fibronectin | F | GAGAATAAGCTGTACCATCGCAA |
|  | R | CGACCACATAGGAAGTCCCAG |
| GAPDH | F | AACTTTGGTATCGTGGAAGG |
|  | R | GCCAGTAGAGGCAGGGATGA |
