# Supplementary material for: GPR65 inhibits human trophoblast cell adhesion through upregulation of MYLK and downregulation of fibronectin via cAMP-ERK signaling in a low pH environment
Source: Cell Commun Signal. 2023 Sep 18;21:238. doi: 10.1186/s12964-023-01249-3 (PMC10506227; doi:10.1186/s12964-023-01249-3)
Supplement: Supplementary file 3 — Additional file 2. [file 12964_2023_1249_MOESM2_ESM.docx]

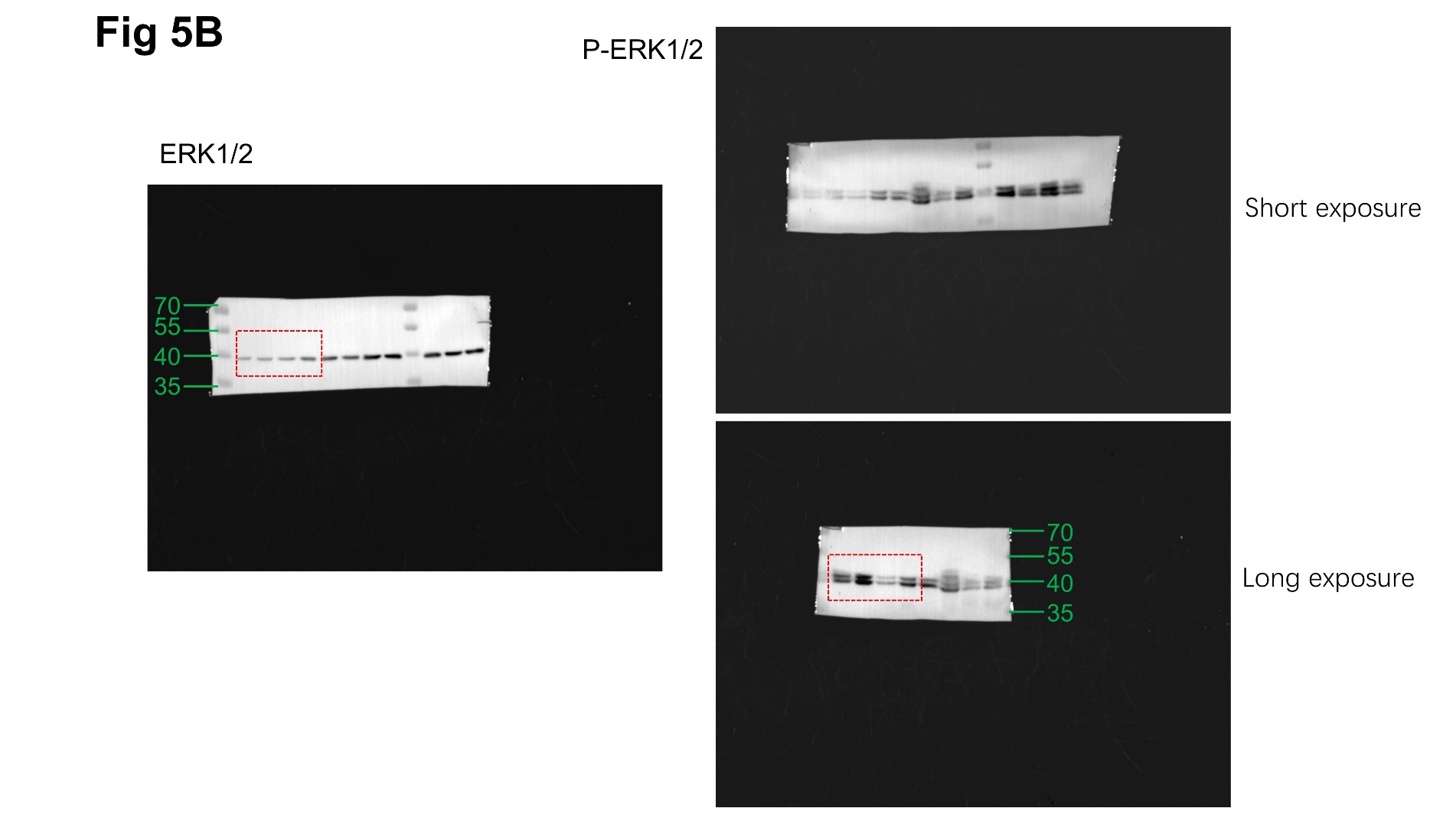


Figure 5-source data 1

Original blots of Figure 5B.


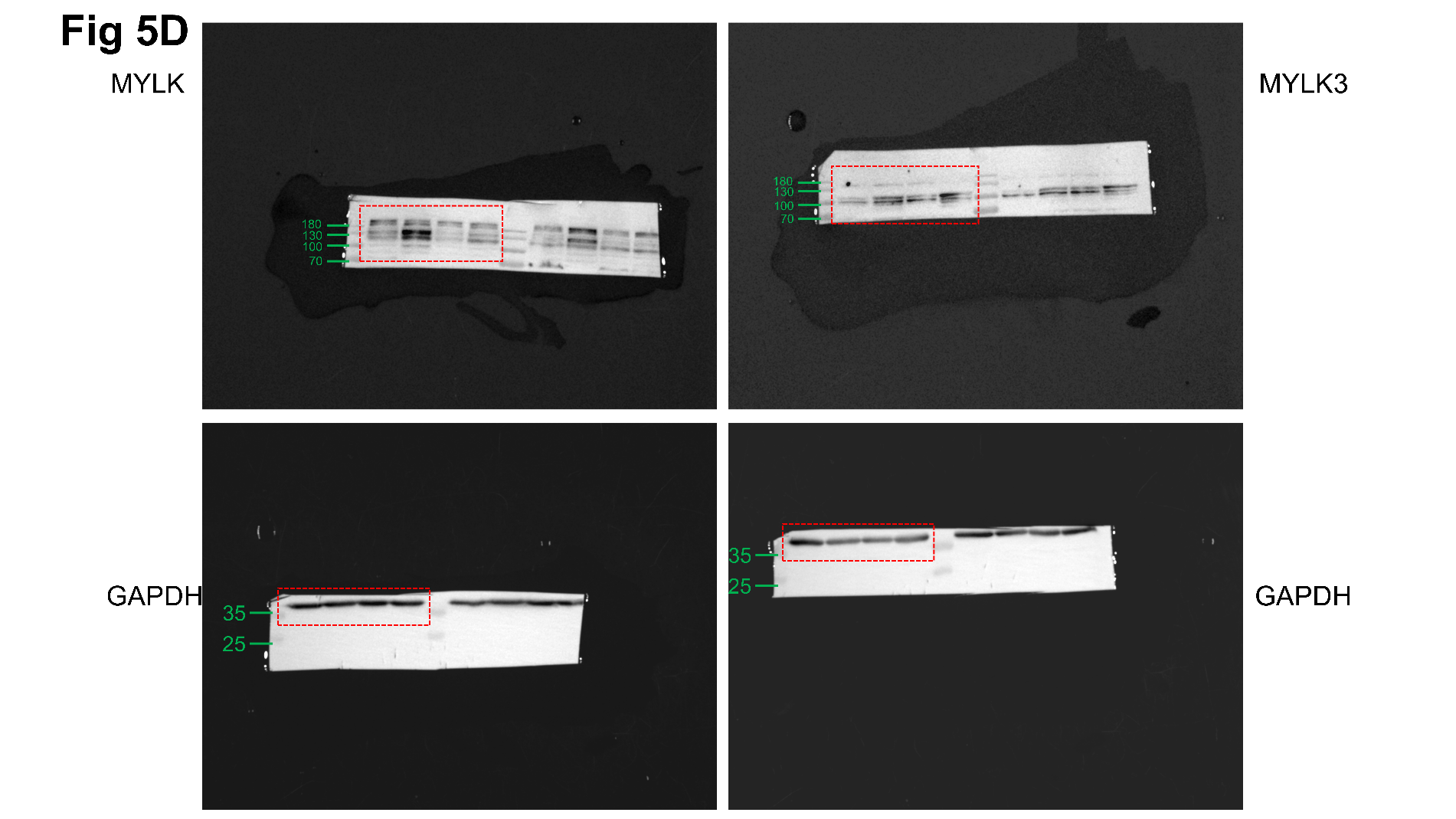


Figure 5-source data 2

Original blots of Figure 5D.


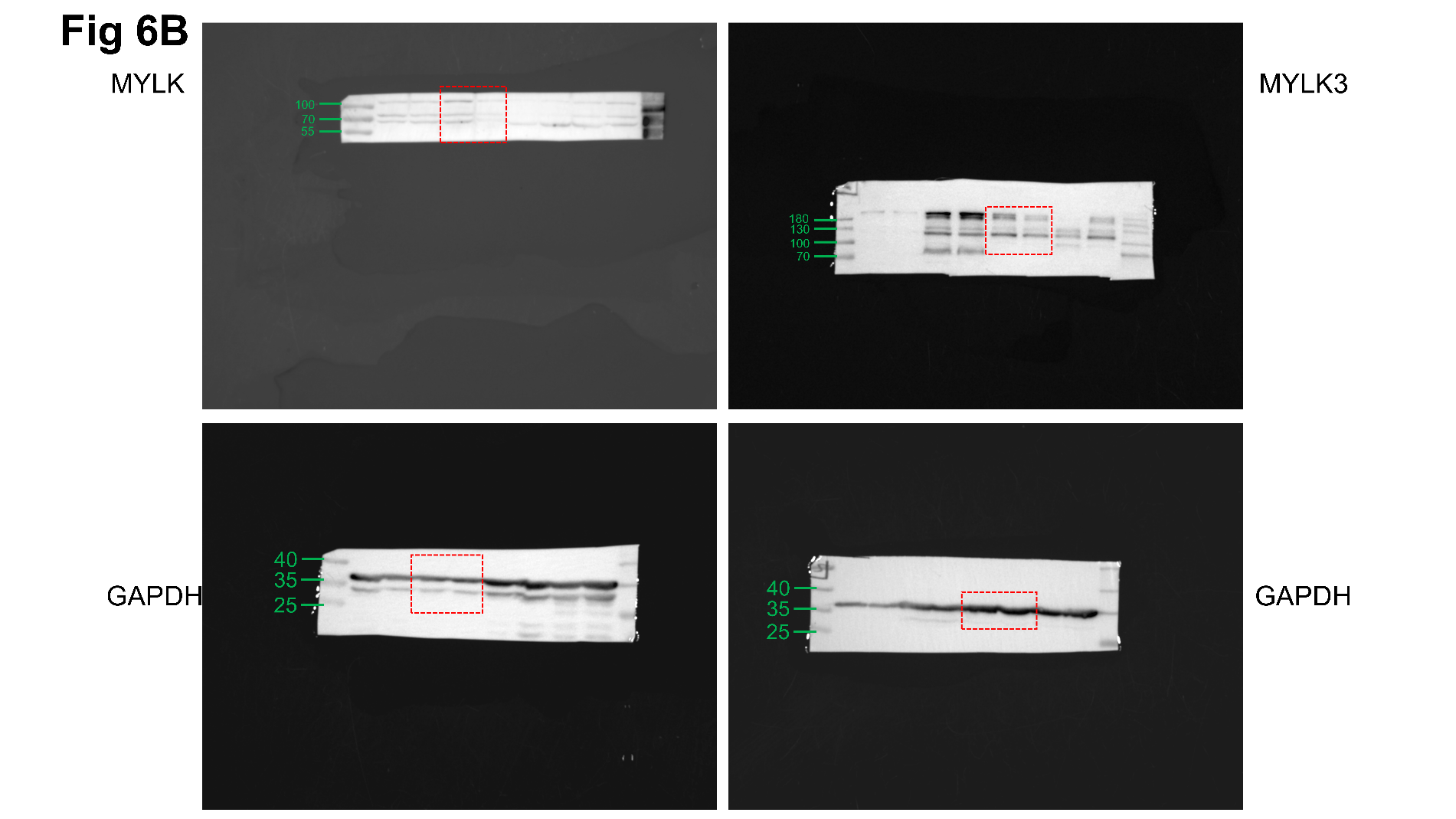
Figure 6-source data 1

Original blots of Figure 6B.


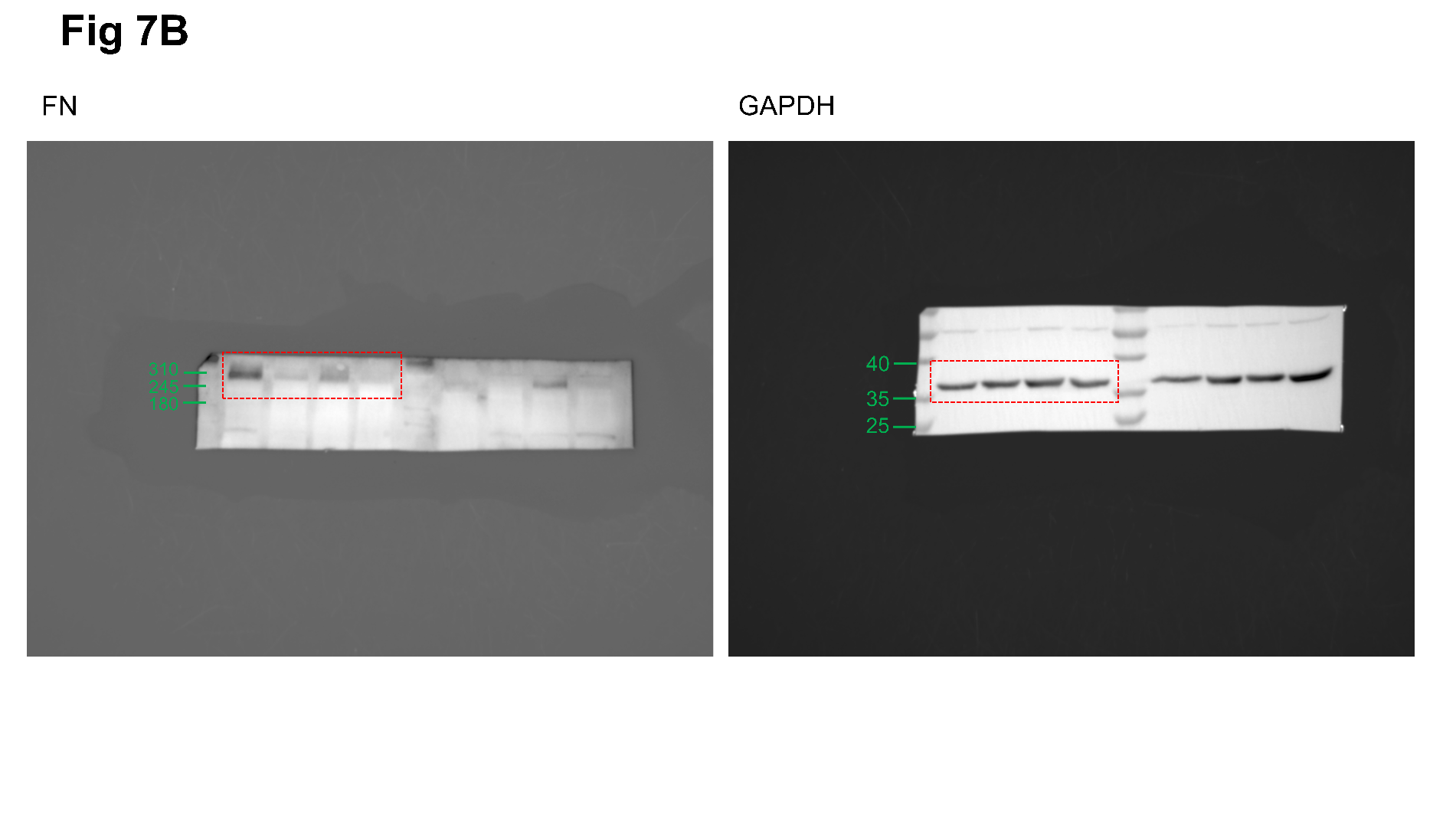


Figure 7-source data 1

Original blots of Figure 7B.


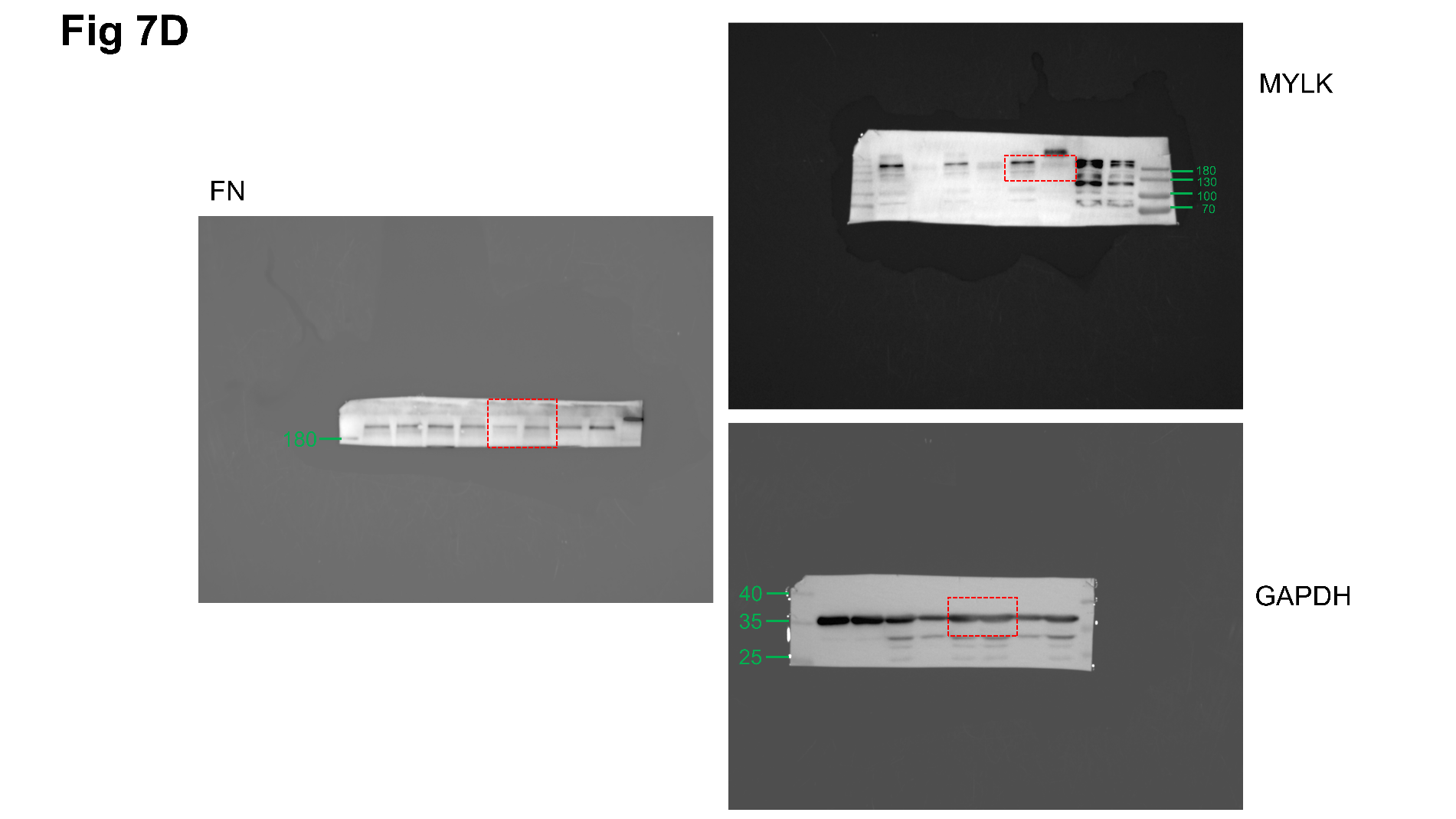


Figure 7-source data 2

Original blots of Figure 7D.


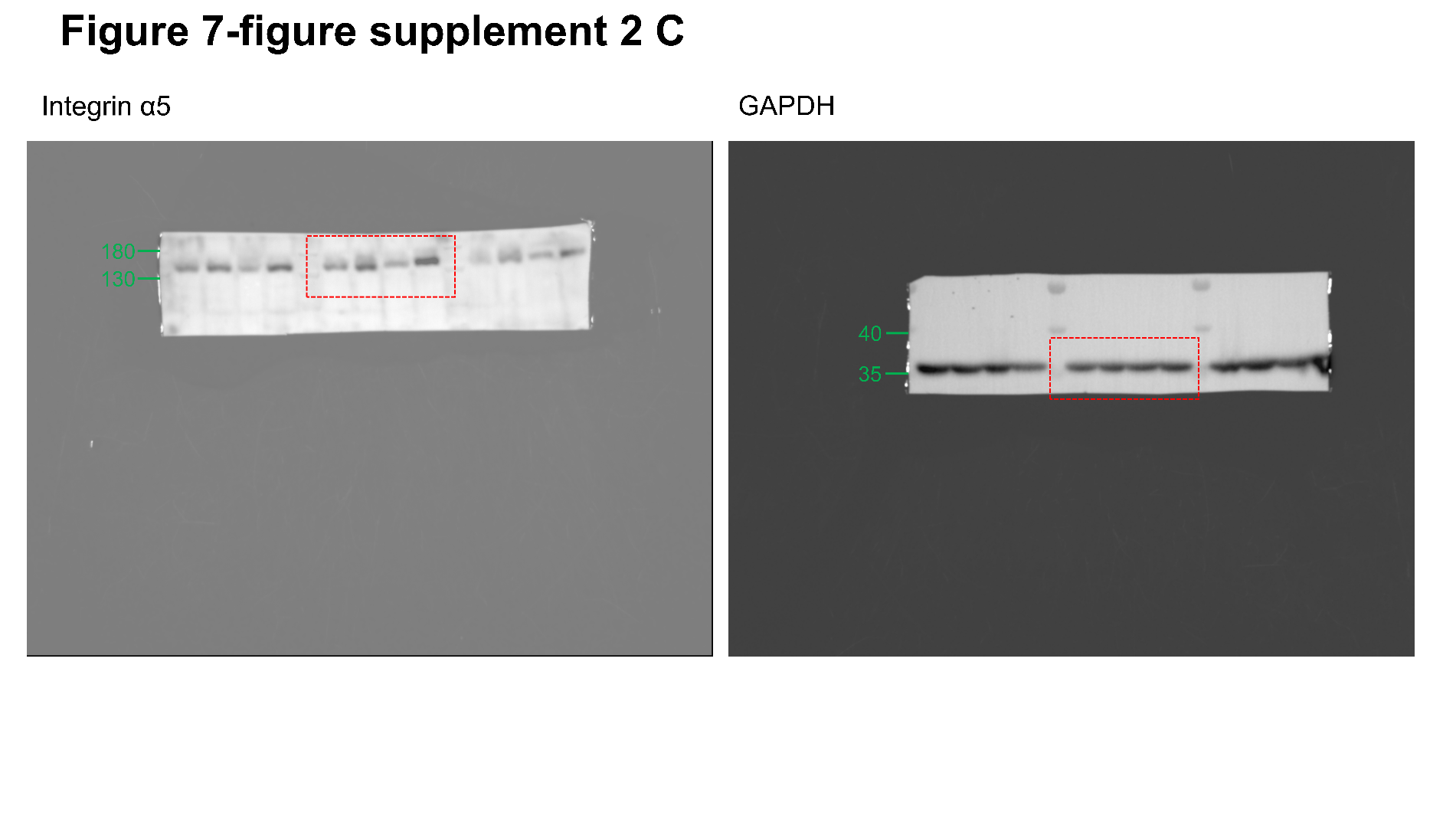


Figure 7-figure supplement 2

Original blots of Figure 7- figure supplement 2 C.
